# Supplementary material for: A generalized theoretical framework to investigate multicomponent actin dynamics
Source: PLoS Comput Biol. 2025 Sep 8;21(9):e1013434. doi: 10.1371/journal.pcbi.1013434 (PMC12431664; doi:10.1371/journal.pcbi.1013434)
Supplement: S1 Text — Includes detailed derivations of statistical moments for the general kinetic model, illustrative examples, procedures for error estimation, and the methodology used for analyzing experimental data. Includes Fig A, showing the schematics of the matrices 𝐑^, 𝐊^, 𝐏(ΔLt), and the decomposition of the transition probability vector. (PDF) [file pcbi.1013434.s001.pdf]

Supporting information
for
A generalized theoretical framework to investigate multicomponent actin dynamics

Mintu Nandi, Shashank Shekhar, Sandeep Choubey

1 Moments of actin filament length distribution in the presence of an arbitrary number of actin binding proteins

To compute all the moments of 1) filament length distributions as a function of time and 2) the long-time distribution of filament lengths within a fixed time window, we use the master equation (1) given in the main text given in the main text, as described in the main text. This equation can be exactly solved to obtain the n-th moment of the distribution of ΔL_t as a function of time and ΔL_τ. To compute the transient moments, we define the following vectors of partial moments,

$$\begin{aligned}\overrightarrow{\Delta L}_t^{(0)} &= \sum_{\Delta L_t=-\infty}^{\infty} \Delta L_t^0 \mathbf{P}(\Delta L_t) \\ &= \left(\sum_{\Delta L_t=-\infty}^{\infty} P_1(\Delta L_t), \ \cdots, \ \sum_{\Delta L_t=-\infty}^{\infty} P_{N_1}(\Delta L_t), \ \sum_{\Delta L_t=-\infty}^{\infty} P_{N_1+1}(\Delta L_t), \ \cdots, \ \sum_{\Delta L_t=-\infty}^{\infty} P_N(\Delta L_t) \right)^T, \end{aligned} \tag{1}$$

$$\begin{aligned}\overrightarrow{\Delta L}_t^{(n)} &= \sum_{\Delta L_t=-\infty}^{\infty} \Delta L_t^n \mathbf{P}(\Delta L_t) \\ &= \left(\sum_{\Delta L_t=-\infty}^{\infty} \Delta L_t^n P_1(\Delta L_t), \ \cdots, \ \sum_{\Delta L_t=-\infty}^{\infty} \Delta L_t^n P_{N_1}(\Delta L_t), \ \sum_{\Delta L_t=-\infty}^{\infty} \Delta L_t^n P_{N_1+1}(\Delta L_t), \ \cdots, \right. \\ &\quad \left. \sum_{\Delta L_t=-\infty}^{\infty} \Delta L_t^n P_N(\Delta L_t) \right)^T. \end{aligned} \tag{2}$$

In Eq. (1), $\sum_{\Delta L_t=-\infty}^{\infty} P_i(\Delta L_t)$ represents the probability of occurrence of filament state i (i.e., state visit probability). In Eq. (2), $\sum_{\Delta L_t=-\infty}^{\infty} \Delta L_t^n P_i(\Delta L_t)$ stands for the nth partial moment due to the state i. These vectors are instrumental in calculating the moments of the probability distribution of the change in length of the filament. For instance, the nth central moment can be expressed as the sum of all elements of the vector $\overrightarrow{\Delta L}_t^{(n)}$, i.e.,

$$\langle \Delta L_t^n \rangle = \sum_{i=1}^{N} \left(\overrightarrow{\Delta L}_t^{(n)} \right)_{i1} = \overrightarrow{Y}.\overrightarrow{\Delta L}_t^{(n)}, \tag{3}$$

where $\overrightarrow{Y} = (1, \ 1, \ \cdots, \ 1)_{(1\times N)}$. Here, $\overrightarrow{Y} \cdot \overrightarrow{\Delta L}_t^{(0)} = 1$, as the total state visit probability must always equal unity. We note that Eq. (3) sums the contributions from all states in the nth partial moment vector $\overrightarrow{\Delta L}_t^{(n)}$ (Eq. (2)) to compute the total moment exactly, without any approximation.

To calculate $\overrightarrow{\Delta L}_t^{(n)}$, both sides of master equation (1) given in the main text are multiplied by ΔL_t^n, followed by summing over all values of ΔL_t, which results in

$$\begin{aligned}\frac{d}{dt}\overrightarrow{\Delta L}_t^{(n)} &= \sum_{\Delta L_t=-\infty}^{\infty} \Delta L_t^n \frac{d}{dt}\mathbf{P}(\Delta L_t), \\ &= \left(\hat{\mathbf{K}} - \hat{\mathbf{R}}\right) \overrightarrow{\Delta L}_t^{(n)} + \hat{\mathbf{R}} \left[\sum_{\Delta L_t=-\infty}^{\infty} (1+\Delta L_t)^n \mathbf{P}_\uparrow(\Delta L_t) + \sum_{\Delta L_t=-\infty}^{\infty} (-1)^n (1-\Delta L_t)^n \mathbf{P}_\downarrow(\Delta L_t) \right]. \end{aligned} \tag{4}$$

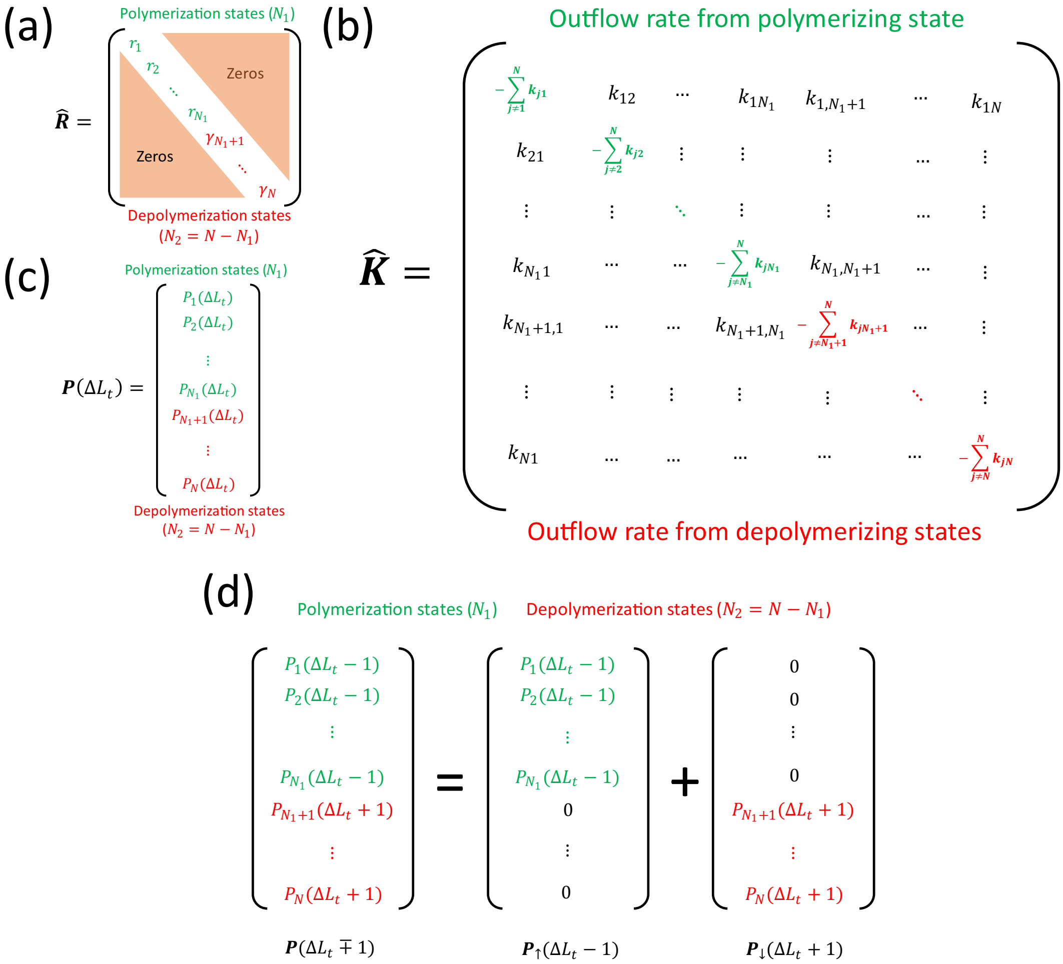

Figure A: The schematics of structures of the matrices (a) $\hat{\mathbf{R}}$, (b) $\hat{\mathbf{K}}$, and (c) $\mathbf{P}(\Delta L_t)$. (d) The decomposition of transition probability vector.

In deriving Eq. (4), we have employed the change of variable $\Delta L_t - 1 \to \Delta L_t'$ and $\Delta L_t + 1 \to \Delta L_t''$ followed by revert back to ΔL_t for notational simplicity. We define the binomial expansions $(1 + \Delta L_t)^n = \sum_{x=0}^{n} \binom{n}{x} \Delta L_t^x = 1 + \sum_{x=1}^{n} \binom{n}{x} \Delta L_t^x$ and $(1 - \Delta L_t)^n = \sum_{x=0}^{n} (-1)^x \binom{n}{x} \Delta L_t^x = 1 + \sum_{x=1}^{n} (-1)^x \binom{n}{x} \Delta L_t^x$, where $\binom{n}{x} = n!/(x!(n-x)!)$ is called binomial coefficient. Employing the binomial expansion on Eq. (4) yields,

$$\begin{aligned}\frac{d}{dt}\overrightarrow{\Delta L}_t^{(n)} &= \left(\hat{\mathbf{K}} - \hat{\mathbf{R}}\right)\overrightarrow{\Delta L}_t^{(n)} + \hat{\mathbf{R}} \sum_{\Delta L_t = -\infty}^{\infty} \left(1 + \sum_{x=1}^{n} \binom{n}{x} \Delta L_t^x\right) \mathbf{P}_\uparrow(\Delta L_t) \\ &+ \hat{\mathbf{R}} \sum_{\Delta L_t = -\infty}^{\infty} (-1)^n \left(1 + \sum_{x=1}^{n} (-1)^x \binom{n}{x} \Delta L_t^x\right) \mathbf{P}_\downarrow(\Delta L_t). \end{aligned} \tag{5}$$

We now define $\overrightarrow{\Delta L}_{t_\uparrow}^{(0)} = \sum_{\Delta L_t=-\infty}^{\infty} \mathbf{P}_\uparrow(\Delta L_t)$ and $\overrightarrow{\Delta L}_{t_\downarrow}^{(0)} = \sum_{\Delta L_t=-\infty}^{\infty} \mathbf{P}_\downarrow(\Delta L_t)$ as the state visit probabilities from polymerizing and depolymerizing states, respectively. This gives $\overrightarrow{\Delta L}_t^{(0)} = \overrightarrow{\Delta L}_{t_\uparrow}^{(0)} + \overrightarrow{\Delta L}_{t_\downarrow}^{(0)}$. Again, the nth partial moment vector follow the similar relation, i.e., $\overrightarrow{\Delta L}_t^{(n)} = \overrightarrow{\Delta L}_{t_\uparrow}^{(n)} + \overrightarrow{\Delta L}_{t_\downarrow}^{(n)}$, where partial moment vector due to polymerizing and depolymerizing states are defined as, $\overrightarrow{\Delta L}_{t_\uparrow}^{(n)} = \sum_{\Delta L_t=-\infty}^{\infty} \Delta L_t^n \mathbf{P}_\uparrow(\Delta L_t)$ and $\overrightarrow{\Delta L}_{t_\downarrow}^{(n)} = \sum_{\Delta L_t=-\infty}^{\infty} \Delta L_t^n \mathbf{P}_\downarrow(\Delta L_t)$, respectively. Using these definitions, we rearrange Eq. (5) as,

$$\frac{d}{dt}\overrightarrow{\Delta L}_t^{(n)} = \hat{\mathbf{K}}\overrightarrow{\Delta L}_t^{(n)} + \hat{\mathbf{R}}\left[\overrightarrow{\Delta L}_{t_\uparrow}^{(0)} + (-1)^n \overrightarrow{\Delta L}_{t_\downarrow}^{(0)}\right] + \hat{\mathbf{R}} \sum_{x=1}^{n-1} \binom{n}{x} \left[\overrightarrow{\Delta L}_{t_\uparrow}^{(x)} + (-1)^{n+x} \overrightarrow{\Delta L}_{t_\downarrow}^{(x)}\right]. \tag{6}$$

We multiply both sides of Eq. (6) by $\overrightarrow{Y}$ and use Eq. (3), which leads to

$$\frac{d}{dt}\langle \Delta L_t^n \rangle = \overrightarrow{R}\left[\overrightarrow{\Delta L}_{t_\uparrow}^{(0)} + (-1)^n \overrightarrow{\Delta L}_{t_\downarrow}^{(0)}\right] + \overrightarrow{R}\sum_{x=1}^{n-1}\binom{n}{x}\left[\overrightarrow{\Delta L}_{t_\uparrow}^{(x)} + (-1)^{n+x}\overrightarrow{\Delta L}_{t_\downarrow}^{(x)}\right], \tag{7}$$

where, following the definition of matrices $\hat{\mathbf{K}}$ and $\hat{\mathbf{R}}$, we arrive at $\overrightarrow{Y}.\hat{\mathbf{K}} = 0$ and $\overrightarrow{R} = \overrightarrow{Y}.\hat{\mathbf{R}}$ where $\overrightarrow{R}$ represents a row vector containing the diagonal elements of $\hat{\mathbf{R}}$. Eq. (7) forms the nth moment equation. Solving Eq. (7) yields the expression of nth central moment for ΔL_t, provided we have the explicit expressions of $\overrightarrow{\Delta L}_{t_\uparrow}^{(0)}$, $\overrightarrow{\Delta L}_{t_\downarrow}^{(0)}$, $\overrightarrow{\Delta L}_{t_\uparrow}^{(x)}$, and $\overrightarrow{\Delta L}_{t_\downarrow}^{(x)}$. To solve this moment equation, we utilize the Laplace transformation with the initial condition that $\langle \Delta L_{t=0}^n \rangle = 0$, reflecting the fact that filament elongation or shortening has not started yet at initial times. This approach leads to the following expression

$$\langle \Delta L_s^n \rangle = \frac{1}{s}\overrightarrow{R}\left[\overrightarrow{\Delta L}_{s_\uparrow}^{(0)} + (-1)^n \overrightarrow{\Delta L}_{s_\downarrow}^{(0)}\right] + \frac{1}{s}\overrightarrow{R}\sum_{x=1}^{n-1}\binom{n}{x}\left[\overrightarrow{\Delta L}_{s_\uparrow}^{(x)} + (-1)^{n+x}\overrightarrow{\Delta L}_{s_\downarrow}^{(x)}\right]. \tag{8}$$

To find $\overrightarrow{\Delta L}_{s_\uparrow}^{(0)}$ and $\overrightarrow{\Delta L}_{s_\downarrow}^{(0)}$ associated with Eq.(8), we begin by computing the equation for $\overrightarrow{\Delta L}_{t}^{(0)}$ by summing master equation (1) given in the main text over all values of ΔL_t. This results in

$$\frac{d}{dt}\overrightarrow{\Delta L}_{t}^{(0)} = \sum_{\Delta L_t=-\infty}^{\infty}\frac{d}{dt}\mathbf{P}(\Delta L_t) = \hat{\mathbf{K}}\overrightarrow{\Delta L}_{t}^{(0)}. \tag{9}$$

While deriving Eq. (9), we use the change of variable $\Delta L_t - 1 \to \Delta L_t'$ and $\Delta L_t + 1 \to \Delta L_t''$. Solving Eq. (9) in Laplace domain with the initial condition that at the initial time, the filament exists in state 1 (free state) which yields $\overrightarrow{\Delta L}_{t=0}^{(0)} = (1\ 0\ \cdots\ 0)^T = \overrightarrow{I}$. This operation results in,

$$\begin{aligned}\overrightarrow{\Delta L}_{s}^{(0)} &= (s\hat{\mathbf{I}} - \hat{\mathbf{K}})^{-1}\overrightarrow{I},\\ \overrightarrow{\Delta L}_{s_\uparrow}^{(0)} + \overrightarrow{\Delta L}_{s_\downarrow}^{(0)} &= (s\hat{\mathbf{I}} - \hat{\mathbf{K}})^{-1}\overrightarrow{I}\end{aligned} \tag{10}$$

where $\hat{\mathbf{I}}$ stands for the identity matrix. Now, it is important to decompose the vector obtained from the matrix operation of the right-hand side of Eq. (10) and must correspond to the decomposition of $\mathbf{P}(\Delta L_t \mp 1)$ (see Fig A). The decomposition of $(s\hat{\mathbf{I}} - \hat{\mathbf{K}})^{-1}\overrightarrow{I}$ results in two vectors: one has non-zero values of the elements for polymerizing states and zero values for depolymerizing states, which provides the expression of $\overrightarrow{\Delta L}_{s_\uparrow}^{(0)}$. On the contrary, the second vector contains zero values of the elements for polymerizing states and non-zero values for depolymerizing states, resulting in $\overrightarrow{\Delta L}_{s_\downarrow}^{(0)}$. As the general matrix forms of $\overrightarrow{\Delta L}_{s_\uparrow}^{(0)}$ and $\overrightarrow{\Delta L}_{s_\downarrow}^{(0)}$ are difficult to show, the computation of these two vectors has been performed using suitable software (for respective scripts, see GitHub repository link given in *Data Availability*).

To compute $\overrightarrow{\Delta L}_{s_\uparrow}^{(x)}$ and $\overrightarrow{\Delta L}_{s_\downarrow}^{(x)}$, we derive the equation for $\overrightarrow{\Delta L}_{t}^{(x)}$ by rewriting Eq. (6) in terms of xth partial moment equation as,

$$\frac{d}{dt}\overrightarrow{\Delta L}_{t}^{(x)} = \hat{\mathbf{K}}\overrightarrow{\Delta L}_{t}^{(x)} + \hat{\mathbf{R}}\left[\overrightarrow{\Delta L}_{t_\uparrow}^{(0)} + (-1)^n \overrightarrow{\Delta L}_{t_\downarrow}^{(0)}\right] + \hat{\mathbf{R}}\sum_{z=1}^{x-1}\binom{x}{z}\left[\overrightarrow{\Delta L}_{t_\uparrow}^{(z)} + (-1)^{x+z}\overrightarrow{\Delta L}_{t_\downarrow}^{(z)}\right]. \tag{11}$$

Upon Laplace transformation with the initial condition that at $t = 0$ the partial moments are zero, i.e., $\overrightarrow{\Delta L}_{t=0}^{(x)} = 0$, Eq. (11) results in,

$$\begin{aligned}\overrightarrow{\Delta L}_{s}^{(x)} &= (s\hat{\mathbf{I}} - \hat{\mathbf{K}})^{-1}\hat{\mathbf{R}}\left[\overrightarrow{\Delta L}_{s_\uparrow}^{(0)} + (-1)^x \overrightarrow{\Delta L}_{s_\downarrow}^{(0)}\right] + (s\hat{\mathbf{I}} - \hat{\mathbf{K}})^{-1}\hat{\mathbf{R}}\sum_{z=1}^{x-1}\binom{x}{z}\left[\overrightarrow{\Delta L}_{s_\uparrow}^{(z)} + (-1)^{x+z}\overrightarrow{\Delta L}_{s_\downarrow}^{(z)}\right],\\ \overrightarrow{\Delta L}_{s_\uparrow}^{(x)} + \overrightarrow{\Delta L}_{s_\downarrow}^{(x)} &= (s\hat{\mathbf{I}} - \hat{\mathbf{K}})^{-1}\hat{\mathbf{R}}\left[\overrightarrow{\Delta L}_{s_\uparrow}^{(0)} + (-1)^x \overrightarrow{\Delta L}_{s_\downarrow}^{(0)}\right] + (s\hat{\mathbf{I}} - \hat{\mathbf{K}})^{-1}\hat{\mathbf{R}}\sum_{z=1}^{x-1}\binom{x}{z}\left[\overrightarrow{\Delta L}_{s_\uparrow}^{(z)} + (-1)^{x+z}\overrightarrow{\Delta L}_{s_\downarrow}^{(z)}\right],\end{aligned} \tag{12}$$

We know that $\overrightarrow{\Delta L}_{t}^{(x)} = \overrightarrow{\Delta L}_{t_\uparrow}^{(x)} + \overrightarrow{\Delta L}_{t_\downarrow}^{(x)}$ are also true in Laplace domain. Using this equality and following the previously stated decomposition of $\overrightarrow{\Delta L}_{t}^{(0)}$, we compute the right-hand side of Eq. (12), which yields a vector of dimension $N \times 1$. We then decompose this vector into polymerizing and depolymerizing components: in the polymerizing vector, the first N_1 elements are non-zero and the remaining N_2 elements are zero; conversely, in the depolymerizing

vector, the first N_1 elements are set to zero and the next N_2 elements are non-zero. This method of decomposition yields $\overrightarrow{\Delta L}^{(x)}_{t_\uparrow}$ and $\overrightarrow{\Delta L}^{(x)}_{t_\downarrow}$.

Eq. (8) together with Eqs. (10) and (12) compute the expression of nth central moment in the Laplace domain. We write the expression of time-dependent n^{th} central moment of the change in length of the filament by performing inverse Laplace transformation on Eq. (8), which gives

$$\langle \Delta L^n_t \rangle = \overrightarrow{R}\left[\overrightarrow{\Lambda}^{(0)}_{t_\uparrow} + (-1)^n \overrightarrow{\Lambda}^{(0)}_{t_\downarrow}\right] + \overrightarrow{R}\sum_{x=1}^{n-1}\binom{n}{x}\left[\overrightarrow{\Lambda}^{(x)}_{t_\uparrow} + (-1)^{n+x}\overrightarrow{\Lambda}^{(x)}_{t_\downarrow}\right], \tag{13}$$

where, $\overrightarrow{\Lambda}^{(0)}_{t_\uparrow} = \mathcal{L}^{-1}\left[\overrightarrow{\Delta L}^{(0)}_{s_\uparrow}/s\right]$, $\overrightarrow{\Lambda}^{(0)}_{t_\downarrow} = \mathcal{L}^{-1}\left[\overrightarrow{\Delta L}^{(0)}_{s_\downarrow}/s\right]$, $\overrightarrow{\Lambda}^{(x)}_{t_\uparrow} = \mathcal{L}^{-1}\left[\overrightarrow{\Delta L}^{(x)}_{s_\uparrow}/s\right]$, and $\overrightarrow{\Lambda}^{(x)}_{t_\downarrow} = \mathcal{L}^{-1}\left[\overrightarrow{\Delta L}^{(x)}_{s_\downarrow}/s\right]$. Here, the symbol $\mathcal{L}^{-1}$ is used to refer the inverse Laplace transformation.

To compute the moments of actin length distribution in long-time limit, we begin by setting the left-hand side of Eq. (9) to zero as the probabilities of visiting different states become independent of time and we denote it as $\overrightarrow{\Delta L}^{(0)}$. Additionally, the sum of state visit probabilities will be unity in this limit. Mathematically, we express these two conditions as,

$$\hat{\mathbf{K}}.\overrightarrow{\Delta L}^{(0)} = 0, \tag{14}$$

$$\overrightarrow{Y}.\overrightarrow{\Delta L}^{(0)} = 1. \tag{15}$$

We note that the long-time condition is closely analogous, though not equivalent, to finding the steady-state from a standard Markov matrix. Our model is based on a continuous-time master equation with a rate matrix $\hat{\mathbf{K}}$, whose columns sums to zero, in contrast to the column normalized transition matrix in discrete-time Markov chain. On solving Eqs. (14) and (15), the state visit probability vector $\overrightarrow{\Delta L}^{(0)}$ is computed. We decompose $\overrightarrow{\Delta L}^{(0)}$ into two partial vectors: one is the polymerizing vector, $\overrightarrow{\Delta L}^{(0)}_{\uparrow}$, where the first N_1 elements are non-zero and the remaining N_2 elements are zero, and second the depolymerizing vector $\overrightarrow{\Delta L}^{(0)}_{\downarrow}$, where the first N_1 elements are zero and the next N_2 elements are non-zero. We have used this method of decomposition in the case of deriving temporal moments.

We now replace the time variable t with τ in Eq. (7) and perform Laplace transformation on both sides of the equation with the initial condition that $\langle \Delta L^n_{t\to\infty}\rangle\big|_\tau = 0$. This initial condition is chosen to track the change in length of the filament that occurs within the window from t to $t+\tau$, excluding any change in length that occurred up to time t. These operations lead to

$$\langle \Delta L^n_s \rangle\big|_\tau = \frac{1}{s^2}\overrightarrow{R}\left[\overrightarrow{\Delta L}^{(0)}_{\uparrow} + (-1)^n \overrightarrow{\Delta L}^{(0)}_{\downarrow}\right] + \frac{1}{s}\overrightarrow{R}\sum_{x=1}^{n-1}\binom{n}{x}\left[\overrightarrow{\Delta L}^{(x)}_{s_\uparrow}\big|_\tau + (-1)^{n+x}\overrightarrow{\Delta L}^{(x)}_{s_\downarrow}\big|_\tau\right], \tag{16}$$

where, the terms $\overrightarrow{\Delta L}^{(0)}_{\uparrow}$ and $\overrightarrow{\Delta L}^{(0)}_{\downarrow}$ are known from Eqs. (14-15). The unknown quantities $\overrightarrow{\Delta L}^{(x)}_{s_\uparrow}\big|_\tau$ and $\overrightarrow{\Delta L}^{(x)}_{s_\downarrow}\big|_\tau$ are computed by evaluating Eq. (11) in the long-time limit and we then perform Laplace transformation with the initial condition that $\overrightarrow{\Delta L}^{(x)}_{t\to\infty}\big|_\tau = 0$. This results in,

$$\begin{aligned}
\overrightarrow{\Delta L}^{(x)}_{s}\big|_\tau &= \frac{1}{s}(s\hat{\mathbf{I}} - \hat{\mathbf{K}})^{-1}\hat{\mathbf{R}}\left[\overrightarrow{\Delta L}^{(0)}_{\uparrow} + (-1)^x \overrightarrow{\Delta L}^{(0)}_{\downarrow}\right] \\
&\quad + (s\hat{\mathbf{I}} - \hat{\mathbf{K}})^{-1}\hat{\mathbf{R}}\sum_{z=1}^{x-1}\binom{x}{z}\left[\overrightarrow{\Delta L}^{(z)}_{s_\uparrow}\big|_\tau + (-1)^{x+z}\overrightarrow{\Delta L}^{(z)}_{s_\downarrow}\big|_\tau\right], \\
\overrightarrow{\Delta L}^{(x)}_{s_\uparrow}\big|_\tau + \overrightarrow{\Delta L}^{(x)}_{s_\downarrow}\big|_\tau &= \frac{1}{s}(s\hat{\mathbf{I}} - \hat{\mathbf{K}})^{-1}\hat{\mathbf{R}}\left[\overrightarrow{\Delta L}^{(0)}_{\uparrow} + (-1)^x \overrightarrow{\Delta L}^{(0)}_{\downarrow}\right] \\
&\quad + (s\hat{\mathbf{I}} - \hat{\mathbf{K}})^{-1}\hat{\mathbf{R}}\sum_{z=1}^{x-1}\binom{x}{z}\left[\overrightarrow{\Delta L}^{(z)}_{s_\uparrow}\big|_\tau + (-1)^{x+z}\overrightarrow{\Delta L}^{(z)}_{s_\downarrow}\big|_\tau\right].
\end{aligned} \tag{17}$$

The decomposition of partial moment vector also applies to long-time scenario. Using this decomposition, we have $\overrightarrow{\Delta L}^{(x)}_{s}\big|_\tau = \overrightarrow{\Delta L}^{(x)}_{s_\uparrow}\big|_\tau + \overrightarrow{\Delta L}^{(x)}_{s_\downarrow}\big|_\tau$. We now decompose the resultant vector obtained from the right-hand side of Eq. (17), yielding two partial vectors: one corresponds to $\overrightarrow{\Delta L}^{(x)}_{s_\uparrow}\big|_\tau$ where the first N_1 elements are non-zero but the next N_2 elements are zero, and the second $\overrightarrow{\Delta L}^{(x)}_{s_\downarrow}\big|_\tau$, where the first N_1 elements are zero but the next N_2 elements are non-zero. Performing inverse Laplace transform of Eq. (16) with the help of Eq. (17), we have the nth central moment

as a function of time window τ,

$$\langle \Delta L_\tau^n \rangle = \overrightarrow{R}\left[\overrightarrow{\Delta L}_{\uparrow}^{(0)} + (-1)^n \overrightarrow{\Delta L}_{\downarrow}^{(0)}\right]\tau + \overrightarrow{R}\sum_{x=1}^{n-1}\binom{n}{x}\left[\overrightarrow{\Lambda}_{\tau\uparrow}^{(x)} + (-1)^{n+x}\overrightarrow{\Lambda}_{\tau\downarrow}^{(x)}\right], \tag{18}$$

where, $\overrightarrow{\Lambda}_{\tau\uparrow}^{(x)} = \mathcal{L}^{-1}\left[\frac{\overrightarrow{\Delta L}_{s\uparrow}^{(x)}\big|_\tau}{s}\right]$ and $\overrightarrow{\Lambda}_{\tau\downarrow}^{(x)} = \mathcal{L}^{-1}\left[\frac{\overrightarrow{\Delta L}_{s\downarrow}^{(x)}\big|_\tau}{s}\right]$.

Note that such master equation-based models have been employed in other fields of single-molecule biology to study their governing principles [1, 2, 3].

2 Moments of actin length distribution regulated by an elongator

Elongator molecules, such as formin, bind to the barbed ends of actin filaments, accelerating the polymerization rate. In the presence of formin, the barbed end can exist in two states: the free state, B (state 1), where the barbed end is free, and the formin-bound state, BF (state 2). The rate of formin binding (B→BF) and unbinding (BF→B) are denoted by $k_{21} = k_F^+$ and $k_{12} = k_F^-$, respectively. The polymerization rate from the free barbed end B is r_1, and from the formin-bound barbed end Bf is r_2. The governing master equations are,

$$\frac{dP_B(\Delta L_t)}{dt} = -k_F^+ P_B(\Delta L_t) + k_F^- P_{BF}(\Delta L_t) - r_1 P_B(\Delta L_t) + r_1 P_B(\Delta L_t - 1), \tag{19}$$

$$\frac{dP_{BF}(\Delta L_t)}{dt} = k_F^+ P_B(\Delta L_t) - k_F^- P_{BF}(\Delta L_t) - r_2 P_{BF}(\Delta L_t) + r_2 P_{BF}(\Delta L_t - 1). \tag{20}$$

Expressing the above two equations in matrix form according to the master equation (1) given in the main text, we have,

$$\frac{d}{dt}\mathbf{P}(\Delta L_t) = (\hat{\mathbf{K}} - \hat{\mathbf{R}})\mathbf{P}(\Delta L_t) + \hat{\mathbf{R}}\mathbf{P}(\Delta L_t - 1), \tag{21}$$

where, $\mathbf{P}(\Delta L_t) = \begin{pmatrix} P_B(\Delta L_t) \\ P_{BF}(\Delta L_t) \end{pmatrix}$, $\hat{\mathbf{K}} = \begin{pmatrix} -k_F^+ & k_F^- \\ k_F^+ & -k_F^- \end{pmatrix}$, and $\hat{\mathbf{R}} = \begin{pmatrix} r_1 & 0 \\ 0 & r_2 \end{pmatrix}$. We, now, define the following partial moment vectors as,

$$\overrightarrow{\Delta L}_t^{(0)} = \sum_{\Delta L_t=-\infty}^{\infty}\mathbf{P}(\Delta L_t) = \begin{pmatrix} \sum_{\Delta L_t=-\infty}^{\infty} P_B(\Delta L_t) \\ \sum_{\Delta L_t=-\infty}^{\infty} P_{BF}(\Delta L_t) \end{pmatrix},$$

$$\overrightarrow{\Delta L}_t^{(1)} = \sum_{\Delta L_t=-\infty}^{\infty}\Delta L_t^1\mathbf{P}(\Delta L_t) = \begin{pmatrix} \sum_{\Delta L_t=-\infty}^{\infty} \Delta L_t P_B(\Delta L_t) \\ \sum_{\Delta L_t=-\infty}^{\infty} \Delta L_t P_{BF}(\Delta L_t) \end{pmatrix}.,$$

$$\overrightarrow{\Delta L}_t^{(2)} = \sum_{\Delta L_t=-\infty}^{\infty}\Delta L_t^2\mathbf{P}(\Delta L_t) = \begin{pmatrix} \sum_{\Delta L_t=-\infty}^{\infty} \Delta L_t^2 P_B(\Delta L_t) \\ \sum_{\Delta L_t=-\infty}^{\infty} \Delta L_t^2 P_{BF}(\Delta L_t) \end{pmatrix}. \tag{22}$$

Multiplying both sides of Eq. (21) by ΔL_t followed by summing over all values of ΔL_t, we get,

$$\begin{aligned}
\frac{d}{dt}\overrightarrow{\Delta L}_t^{(1)} &= \sum_{\Delta L_t=-\infty}^{\infty}\Delta L_t\frac{d}{dt}\mathbf{P}(\Delta L_t), \\
&= \left(\hat{\mathbf{K}} - \hat{\mathbf{R}}\right)\sum_{\Delta L_t=-\infty}^{\infty}\Delta L_t\mathbf{P}(\Delta L_t) + \hat{\mathbf{R}}\sum_{\Delta L_t=-\infty}^{\infty}\Delta L_t\mathbf{P}(\Delta L_t - 1), \\
&= \left(\hat{\mathbf{K}} - \hat{\mathbf{R}}\right)\overrightarrow{\Delta L}_t^{(1)} + \hat{\mathbf{R}}\sum_{\Delta L'_t=-\infty}^{\infty}(1 + \Delta L'_t)\mathbf{P}(\Delta L'_t), \\
&= \left(\hat{\mathbf{K}} - \hat{\mathbf{R}}\right)\overrightarrow{\Delta L}_t^{(1)} + \hat{\mathbf{R}}\sum_{\Delta L_t=-\infty}^{\infty}(1 + \Delta L_t)\mathbf{P}(\Delta L_t), \\
&= \left(\hat{\mathbf{K}} - \hat{\mathbf{R}}\right)\overrightarrow{\Delta L}_t^{(1)} + \hat{\mathbf{R}}\overrightarrow{\Delta L}_t^{(0)} + \hat{\mathbf{R}}\overrightarrow{\Delta L}_t^{(1)}, \\
&= \hat{\mathbf{K}}\overrightarrow{\Delta L}_t^{(1)} + \hat{\mathbf{R}}\overrightarrow{\Delta L}_t^{(0)}.
\end{aligned} \tag{23}$$

Multiplying both sides of Eq. (23) by $\overrightarrow{Y} = (1, 1)$ yields,

$$\begin{aligned}
\frac{d}{dt}\overrightarrow{Y}\overrightarrow{\Delta L}_t^{(1)} &= \overrightarrow{Y}.\hat{\mathbf{K}}\overrightarrow{\Delta L}_t^{(1)} + \overrightarrow{Y}.\hat{\mathbf{R}}\overrightarrow{\Delta L}_t^{(0)}, \\
\frac{d}{dt}\langle \Delta L_t \rangle &= \overrightarrow{R}\overrightarrow{\Delta L}_t^{(0)},
\end{aligned} \tag{24}$$

where, we have used $\overrightarrow{Y}\overrightarrow{\Delta L}_t^{(1)} = \langle \Delta L_t \rangle$ (see Eq. (3)), $\overrightarrow{Y}.\hat{\mathbf{K}} = 0$ and $\overrightarrow{Y}.\hat{\mathbf{R}} = \overrightarrow{R} = (r_1, r_2)$. The second equality to zero yields from the fact that the sum of all the elements along a column of matrix $\hat{\mathbf{K}}$ is zero. We, again, multiply both sides of Eq. (21) by ΔL_t^2 followed by summing over all values of ΔL_t and obtain,

$$\begin{aligned}
\frac{d}{dt}\overrightarrow{\Delta L}_t^{(2)} &= \sum_{\Delta L_t=-\infty}^{\infty} \Delta L_t^2 \frac{d}{dt}\mathbf{P}(\Delta L_t), \\
&= \left(\hat{\mathbf{K}} - \hat{\mathbf{R}}\right) \sum_{\Delta L_t=-\infty}^{\infty} \Delta L_t^2 \mathbf{P}(\Delta L_t) + \hat{\mathbf{R}} \sum_{\Delta L_t=-\infty}^{\infty} \Delta L_t^2 \mathbf{P}(\Delta L_t - 1), \\
&= \left(\hat{\mathbf{K}} - \hat{\mathbf{R}}\right) \overrightarrow{\Delta L}_t^{(2)} + \hat{\mathbf{R}} \sum_{\Delta L'_t=-\infty}^{\infty} (1 + \Delta L'_t)^2 \mathbf{P}(\Delta L'_t), \\
&= \left(\hat{\mathbf{K}} - \hat{\mathbf{R}}\right) \overrightarrow{\Delta L}_t^{(2)} + \hat{\mathbf{R}} \sum_{\Delta L_t=-\infty}^{\infty} (1 + \Delta L_t^2 + 2\Delta L_t) \mathbf{P}(\Delta L_t), \\
&= \left(\hat{\mathbf{K}} - \hat{\mathbf{R}}\right) \overrightarrow{\Delta L}_t^{(2)} + \hat{\mathbf{R}}\overrightarrow{\Delta L}_t^{(0)} + \hat{\mathbf{R}}\overrightarrow{\Delta L}_t^{(2)} + 2\hat{\mathbf{R}}\overrightarrow{\Delta L}_t^{(1)}, \\
&= \hat{\mathbf{K}}\overrightarrow{\Delta L}_t^{(2)} + 2\hat{\mathbf{R}}\overrightarrow{\Delta L}_t^{(1)} + \hat{\mathbf{R}}\overrightarrow{\Delta L}_t^{(0)}.
\end{aligned} \tag{25}$$

Multiplying both sides of Eq. (25) by $\overrightarrow{Y}$ yields,

$$\begin{aligned}
\frac{d}{dt}\overrightarrow{Y}\overrightarrow{\Delta L}_t^{(2)} &= \overrightarrow{Y}.\hat{\mathbf{K}}\overrightarrow{\Delta L}_t^{(2)} + 2\overrightarrow{Y}.\hat{\mathbf{R}}\overrightarrow{\Delta L}_t^{(1)} + \overrightarrow{Y}.\hat{\mathbf{R}}\overrightarrow{\Delta L}_t^{(0)}, \\
\frac{d}{dt}\langle \Delta L_t^2 \rangle &= \overrightarrow{R}\overrightarrow{\Delta L}_t^{(0)} + 2\overrightarrow{R}\overrightarrow{\Delta L}_t^{(1)},
\end{aligned} \tag{26}$$

Eqs. (24) and (26) represent the equations for the first and second moments, respectively, which can also be derived from the general moment equation (7). However, when employing this general approach, it's crucial to remember that the state vectors associated with depolymerizing states are zero. To obtain the expressions of the first and, we perform Laplace transformations of Eqs. (24) and (26) with the initial condition that $\Delta L_{t=0} = 0$ (no change in filament length at initial time), which yields,

$$\langle \Delta L_s \rangle = \frac{1}{s}\overrightarrow{R}\overrightarrow{\Delta L}_s^{(0)}, \tag{27}$$

$$\langle \Delta L_s^2 \rangle = \frac{1}{s}\overrightarrow{R}\overrightarrow{\Delta L}_s^{(0)} + \frac{2}{s}\overrightarrow{R}\overrightarrow{\Delta L}_s^{(1)}, \tag{28}$$

Eqs. (27-28) can also be derived from Eq. (8). Now, one needs to find $\overrightarrow{\Delta L}_s^{(0)}$ and $\overrightarrow{\Delta L}_s^{(1)}$ to compute the moments in Laplace space. In order to find $\overrightarrow{\Delta L}_s^{(0)}$, we sum both sides of Eq. (21) over all values of ΔL_t, which results in,

$$\begin{aligned}
\frac{d}{dt}\overrightarrow{\Delta L}_t^{(0)} &= \sum_{\Delta L_t=-\infty}^{\infty} \frac{d}{dt}\mathbf{P}(\Delta L_t), \\
&= \left(\hat{\mathbf{K}} - \hat{\mathbf{R}}\right) \sum_{\Delta L_t=-\infty}^{\infty} \mathbf{P}(\Delta L_t) + \hat{\mathbf{R}} \sum_{\Delta L_t=-\infty}^{\infty} \mathbf{P}(\Delta L_t - 1), \\
&= \left(\hat{\mathbf{K}} - \hat{\mathbf{R}}\right) \overrightarrow{\Delta L}_t^{(0)} + \hat{\mathbf{R}} \sum_{\Delta L'_t=-\infty}^{\infty} \mathbf{P}(\Delta L'_t), \\
&= \left(\hat{\mathbf{K}} - \hat{\mathbf{R}}\right) \overrightarrow{\Delta L}_t^{(0)} + \hat{\mathbf{R}}\overrightarrow{\Delta L}_t^{(0)}, \\
&= \hat{\mathbf{K}}\overrightarrow{\Delta L}_t^{(0)}.
\end{aligned} \tag{29}$$

Now performing Laplace transformation of Eq. (29) with initial condition $\overrightarrow{\Delta L}_{t=0}^{(0)} = (1, 0)^T$, we obtain,

$$\overrightarrow{\Delta L}_s^{(0)} = (s\hat{\mathbf{I}} - \hat{\mathbf{K}})^{-1}\overrightarrow{I}, \tag{30}$$

where, $\hat{\mathbf{I}}$ stands for a 2×2 identity matrix and $\overrightarrow{I} = (1, 0)^T$. To find $\overrightarrow{\Delta L}_s^{(1)}$, we perform Laplace transformation of Eq. (23) with the initial condition that $\overrightarrow{\Delta L}_{t=0}^{(1)} = 0$ to yield,

$$\overrightarrow{\Delta L}_s^{(1)} = (s\hat{\mathbf{I}} - \hat{\mathbf{K}})^{-1}\hat{\mathbf{R}}\overrightarrow{\Delta L}_s^{(0)}, \tag{31}$$

We note that Eq. (31) can also be derived from Eq. (12). Substituting Eqs. (30-31) on Eqs. (27-28) followed by performing inverse Laplace transform from s-space to t-space results in the closed analytical expressions of the moments, $\langle \Delta L_t \rangle$ and $\langle \Delta L_t^2 \rangle$ as a function of time, t. We note that the expressions of these two central moments can also be derived from Eq. (13). We now define the variance of the change in length of the filament as,

$$\sigma^2_{\Delta L_t} = \langle \Delta L_t^2 \rangle - \langle \Delta L_t \rangle^2. \tag{32}$$

After completing the algebraic calculations as discussed, the analytical closed-form solutions for the mean of the change in length of the filament ($\langle \Delta L_t \rangle$) and variance ($\sigma^2_{\Delta L_t}$) are,

$$\langle \Delta L_t \rangle = r_2 t + (r_1 - r_2) A_F t + \frac{(r_1 - r_2)(1 - A_F)}{D_F}(1 - e^{-D_F t}), \tag{33}$$

$$\begin{aligned} \sigma^2_{\Delta L_t} &= r_2 t + (r_1 - r_2) A_F t + \frac{2A_F(1 - A_F)(r_1 - r_2)^2}{D_F} t + \frac{(r_1 - r_2)(1 - A_F)\left[D_F + (r_1 - r_2)(1 - 5A_F)\right]}{D_F^2} \\ &- \left[\frac{(1 - A_F)(r_1 - r_2)}{D_F}\right]^2 e^{-2D_F t} \\ &+ \frac{(1 - A_F)(r_1 - r_2)}{D_F^2}\left[4A_F(r_1 - r_2) - D_F - 2D_F(1 - 2A_F)(r_1 - r_2)t\right] e^{-D_F t}, \end{aligned} \tag{34}$$

where $D_F = k_F^+ + k_F^-$ and $A_F = k_F^-/D_F$.

To compute the mean change in length of the filament and variance at steady state, we begin by considering the state visit probability vector becomes independent of time and we denote it as $\overrightarrow{\Delta L}^{(0)}$. To compute this metric, we set the left-hand side of Eq. (30) to zero to yield $\hat{\mathbf{K}}.\overrightarrow{\Delta L}^{(0)} = 0$. Again, as the total state visit probability is unity, we write $\overrightarrow{Y}.\overrightarrow{\Delta L}^{(0)} = 1$. On solving these two equations, we obtain the expression of the state visit probability vector as,

$$\overrightarrow{\Delta L}^{(0)} = \begin{pmatrix} A_F \\ 1 - A_F \end{pmatrix}. \tag{35}$$

We recast Eqs. (24) and (26) in terms of τ by replacing t and perform Laplace transformation on both sides of each equation with initial conditions, $\langle \Delta L_{t\to\infty} \rangle\big|_\tau = \langle \Delta L^2_{t\to\infty} \rangle\big|_\tau = 0$. These initial conditions are set to capture the change in length of the filament within the time window t to $t + \tau$, excluding any change in length that occurred up to time t. This operation yields,

$$\langle \Delta L_s \rangle\big|_\tau = \frac{1}{s^2}\overrightarrow{R}\overrightarrow{\Delta L}^{(0)}, \tag{36}$$

$$\langle \Delta L_s^2 \rangle\big|_\tau = \frac{1}{s^2}\overrightarrow{R}\overrightarrow{\Delta L}^{(0)} + \frac{2}{s}\overrightarrow{R}\overrightarrow{\Delta L}_s^{(1)}\big|_\tau, \tag{37}$$

To find $\overrightarrow{\Delta L}_s^{(1)}\big|_\tau$ and $\overrightarrow{\Delta L}_s^{(2)}\big|_\tau$, we use Eqs. (23) and (25) for the time window τ and perform Laplace transformation with initial condition that $\overrightarrow{\Delta L}^{(1)}_{t\to\infty}\big|_\tau = 0$ to yield,

$$\overrightarrow{\Delta L}_s^{(1)}\big|_\tau = \frac{1}{s}(s\hat{\mathbf{I}} - \hat{\mathbf{K}})^{-1}\hat{\mathbf{R}}\overrightarrow{\Delta L}^{(0)}, \tag{38}$$

We note that Eqs. (36)-(37) can also be derived from Eq. (16). Additionally, Eq. (38) can also be derived from Eq. (17). Substituting Eqs. (35) and (38) into Eqs. (36)-(37) followed by performing inverse Laplace transformation from s-space to τ-space yield the closed analytical expressions of the central moments, $\langle \Delta L_\tau \rangle$ and $\langle \Delta L_\tau^2 \rangle$ within the time window τ. Using the moments, we define the variance as $\sigma^2_{\Delta L_\tau} = \langle \Delta L_\tau^2 \rangle - \langle \Delta L_\tau \rangle^2$. After performing the algebraic computations, we have,

$$\langle \Delta L_\tau \rangle = r_2 \tau + (r_1 - r_2) A_F \tau, \tag{39}$$

$$\sigma^2_{\Delta L_\tau} = r_2 \tau + (r_1 - r_2) A_F \tau + \frac{2}{D_F}(r_1 - r_2)^2 A_F(1 - A_F)\tau - \frac{2}{D_F^2}(r_1 - r_2)^2 A_F(1 - A_F)(1 - e^{-\tau D_F}). \tag{40}$$

3 Moments of actin length distribution regulated by a capper

The governing master equations for filament length in the presence of a capper is given by

$$\frac{dP_B(\Delta L_t)}{dt} = -k_C^+ P_B(\Delta L_t) + k_C^- P_{BC}(\Delta L_t) - r_1 P_B(\Delta L_t) + r_1 P_B(\Delta L_t - 1), \tag{41}$$

$$\frac{dP_{BC}(\Delta L_t)}{dt} = k_C^+ P_B(\Delta L_t) - k_C^- P_{BC}(\Delta L_t). \tag{42}$$

Expressing the above two equations in matrix form according to Eq. (1), the associated matrices are,
$\mathbf{P}(\Delta L_t) = \begin{pmatrix} P_B(\Delta L_t) \\ P_{BC}(\Delta L_t) \end{pmatrix}$, $\hat{\mathbf{K}} = \begin{pmatrix} -k_C^+ & k_C^- \\ k_C^+ & -k_C^- \end{pmatrix}$, and $\hat{\mathbf{R}} = \begin{pmatrix} r_1 & 0 \\ 0 & 0 \end{pmatrix}$.

Utilizing the general solution provided in Eqs. (13), we derive the corresponding moments (up to the second moment) to obtain $\langle \Delta L_t \rangle$, and $\langle \Delta L_t^2 \rangle$. Using the first and second moments, we express the variance of ΔL_t as $\sigma^2_{\Delta L_t} = \langle \Delta L_t^2 \rangle - \langle \Delta L_t \rangle^2$. The closed-form analytical expressions for mean change in length $\langle \Delta L_t \rangle$, variance $\sigma^2_{\Delta L_t}$ are given by,

$$\langle \Delta L_t \rangle = r_1 A_C t + \frac{r_1(1-A_C)}{D_C}(1-e^{-D_C t}), \tag{43}$$

$$\begin{aligned}\sigma^2_{\Delta L_t} = {} & r_1 A_C t + \frac{r_1(1-A_C)}{D_C}(1-e^{-D_C t}) + \frac{2r_1^2 A_C(1-A_C)t}{D_C} + \left[\frac{r_1(1-A_C)}{D_C}\right]^2 (1-e^{-2D_C t}) \\ & - \frac{2r_1^2(1-A_C)}{D_C^2}(1-e^{-D_C t}) + \frac{2r_1^2(1-A_C)(1-2A_C)}{D_C^2}\left[1-(1+D_C t)e^{-D_C t}\right],\end{aligned} \tag{44}$$

where $D_C = k_C^+ + k_C^-$ and $A_C = k_C^-/D_C$.

Next, we compute the mean, variance, and third central moment in the long-time limit using Eq. (18) and obtain $\langle \Delta L_\tau \rangle$, and $\langle \Delta L_\tau^2 \rangle$. Using first and second central moments, we define the variance as $\sigma^2_{\Delta L_\tau} = \langle \Delta L_\tau^2 \rangle - \langle \Delta L_\tau \rangle^2$. The closed-form expressions of mean and variance are written as,

$$\langle \Delta L_\tau \rangle = r_1 A_C \tau, \tag{45}$$

$$\sigma^2_{\Delta L_\tau} = r_1 A_C \tau + \frac{2}{D_C} r_1^2 A_C(1-A_C)\tau - \frac{2}{D_C^2} r_1^2 A_C (1-A_C)(1-e^{-\tau D_C}). \tag{46}$$

4 Moments of actin length distribution for competitive binding model and simultaneous binding models

4.1 Competitive binding model

In this model, the filament undergoes elongation from states B and BF with rates r_1 and r_2, respectively. To write the master equation according to Eq. (1) given in the main text described in the main text, we define the following matrices,
$\mathbf{P}(\Delta L_t) = \begin{pmatrix} P_B(\Delta L_t) \\ P_{BF}(\Delta L_t) \\ P_{BC}(\Delta L_t) \end{pmatrix}$, $\hat{\mathbf{K}} = \begin{pmatrix} -(k_F^+ + k_C^+) & k_F^- & k_C^- \\ k_F^+ & -k_F^- & 0 \\ k_C^+ & 0 & -k_C^- \end{pmatrix}$, and $\hat{\mathbf{R}} = \begin{pmatrix} r_1 & 0 & 0 \\ 0 & r_2 & 0 \\ 0 & 0 & 0 \end{pmatrix}$.

Using Eq. (13), we derive the corresponding moments $\langle \Delta L_t \rangle$, and $\langle \Delta L_t^2 \rangle$. Using the first and second moments, the variance can be written as $\sigma^2_{\Delta L_t} = \langle \Delta L_t^2 \rangle - \langle \Delta L_t \rangle^2$. Due to the complexity of the analytical expressions, we don't report them here.

Next, we calculate the mean, variance, and third central moment in the long-time limit using Eq. (18) to obtain $\langle \Delta L_\tau \rangle$, $\langle \Delta L_\tau^2 \rangle$, and $\langle \Delta L_\tau^3 \rangle$. Using first and second central moments, we define the variance as $\sigma^2_{\Delta L_\tau} = \langle \Delta L_\tau^2 \rangle - \langle \Delta L_\tau \rangle^2$. The closed form expressions of mean, variance are not reported here due to their complex expressions.

4.2 Simultaneous binding model

As described in the main text, in this model, the filament undergoes elongation from states B and BF with rates r_1 and r_2, respectively. To write the master equation according to Eq. (1) given in the main text described in the main text, we define the following matrices,
$\mathbf{P}(\Delta L_t) = \begin{pmatrix} P_B(\Delta L_t) \\ P_{BF}(\Delta L_t) \\ P_{BC}(\Delta L_t) \\ P_{BFC}(\Delta L_t) \end{pmatrix}$, $\hat{\mathbf{K}} = \begin{pmatrix} -(k_F^+ + k_C^+) & -k_F^- & k_C^- & 0 \\ k_F^+ & -(k_C'^+ + k_F^-) & 0 & k_C'^- \\ k_C^+ & 0 & -(k_F'^+ + k_C^-) & k_F'^- \\ 0 & k_C'^+ & k_F'^+ & -(k_F'^- + k_C'^-) \end{pmatrix}$,
and $\hat{\mathbf{R}} = \begin{pmatrix} r_1 & 0 & 0 & 0 \\ 0 & r_2 & 0 & 0 \\ 0 & 0 & 0 & 0 \\ 0 & 0 & 0 & 0 \end{pmatrix}$.

Using Eq. (13), we derive the corresponding moments $\langle \Delta L_t \rangle$, and $\langle \Delta L_t^2 \rangle$. Using the first and second moments, the variance can be written as $\sigma^2_{\Delta L_t} = \langle \Delta L_t^2 \rangle - \langle \Delta L_t \rangle^2$. The closed form expressions of mean, variance are too cumbersome to be reported here.

Next, we calculate the mean, variance, and third central moment in the long-time limit using Eq. (18) to obtain $\langle \Delta L_\tau \rangle$, and $\langle \Delta L_\tau^2 \rangle$. Using first and second central moments, we define the variance as $\sigma^2_{\Delta L_\tau} = \langle \Delta L_\tau^2 \rangle - \langle \Delta L_\tau \rangle^2$. The closed form expressions of mean, variance are too cumbersome to be reported here.

5 Illustrative examples of simple models of actin length regulations

We review two simplified models of actin filament length regulation under specific conditions. These examples serve to validate the consistency of our model with known kinetic behaviors.

5.1 One-state depolymerization model

We consider a simple one-state model of actin filament which undergoes depolymerization at a rate γ_1, modeled as $\mathrm{B}(L) \xrightarrow{\gamma_1} \mathrm{B}(L-1)$. In this case, the master equation (1) given in the main text simplifies significantly, with no involvement of a transition matrix $\hat{\mathbf{K}}$ and $\mathbf{P}_\uparrow(\Delta L_t - 1)$. The remaining matrices reduce to $\hat{\mathbf{R}} = \gamma_1$, and the probability distributions become $\mathbf{P}(\Delta L_t) = P_B(\Delta L_t)$ and $\mathbf{P}_\uparrow(\Delta L_t - 1) = P_B(\Delta L_t - 1)$.

Using Eq. (13), the transient first and second moments are obtained as:

$$\langle \Delta L_t \rangle = -\gamma_1 t, \tag{47}$$
$$\langle \Delta L_t^2 \rangle = \gamma_1 t + \gamma_1^2 t^2, \tag{48}$$

yielding the variance $\sigma^2_{\Delta L_t} = \gamma_1 t$. Consequently, the Fano factor (Fano $= \sigma^2_{\Delta L_t}/\mathrm{Abs}[\langle \Delta L_t \rangle]$) becomes 1, reflecting Poissonian dynamics.

However, the moments in the long-time limit within the time window τ can be calculated using Eq. (18), which yields,

$$\langle \Delta L_\tau \rangle = -\gamma_1 \tau, \tag{49}$$
$$\langle \Delta L_\tau^2 \rangle = \gamma_1 \tau + \gamma_1^2 \tau^2. \tag{50}$$

The variance $\sigma^2_{\Delta L_\tau} = \gamma_1 \tau$ and the Fano factor becomes 1, reflecting the Poissonian dynamics in long-time limit.

5.2 Two-state polymerization-depolymerization model

We now consider a two-state model consisting of two distinct filament states: B, representing a bare barbed end undergoing polymerization, and B′, representing a depolymerase-bound barbed end undergoing depolymerization. Polymerization and depolymerization occur at rates r_1 and γ_2, respectively. The transition from state B to B′ occurs with rate k_{on}, and the reverse transition with rate k_{off}. The reaction scheme is depicted as:

$$\begin{array}{ccc} \mathrm{B}(L) & \xrightarrow{r_1} & \mathrm{B}(L+1) \\ k_{\mathrm{off}} \uparrow\downarrow k_{\mathrm{on}} & & \\ \mathrm{B}'(L) & \xrightarrow{\gamma_2} & \mathrm{B}'(L-1) \end{array}$$

For this model, the matrices involved in the master equation (1) given in the main text are,

$$\mathbf{P}(\Delta L_t) = \begin{pmatrix} P_B(\Delta L_t) \\ P_{B'}(\Delta L_t) \end{pmatrix}, \hat{\mathbf{K}} = \begin{pmatrix} -k_{\mathrm{on}} & k_{\mathrm{off}} \\ k_{\mathrm{on}} & -k_{\mathrm{off}} \end{pmatrix}, \text{ and } \hat{\mathbf{R}} = \begin{pmatrix} r_1 & 0 \\ 0 & \gamma_2 \end{pmatrix}.$$

Using Eq. (13), we derive the expressions for the first and second moments of the filament length change distribution at time t, under the symmetric condition $k_{\mathrm{on}} = k_{\mathrm{off}} = k$ and $r_1 = \gamma_2 = \omega$. Under these assumptions, the closed-form expressions for the mean and variance are given by,

$$\langle \Delta L_t \rangle = \frac{\omega}{2k}\left(1 - e^{-2kt}\right), \tag{51}$$
$$\sigma^2_{\Delta L_t} = \omega t - \frac{\omega^2}{4k^2}\left[(3 - 4kt) + e^{-2kt}(e^{-2kt} - 4)\right]. \tag{52}$$

In the limit $k \to \infty$, which corresponds to fast state switching between the two states, the mean and variance simplify to $\langle \Delta L_t \rangle = 0$ and $\sigma^2_{\Delta L_t} = \omega t$. This limiting behavior is consistent with a symmetric random walk with net jump

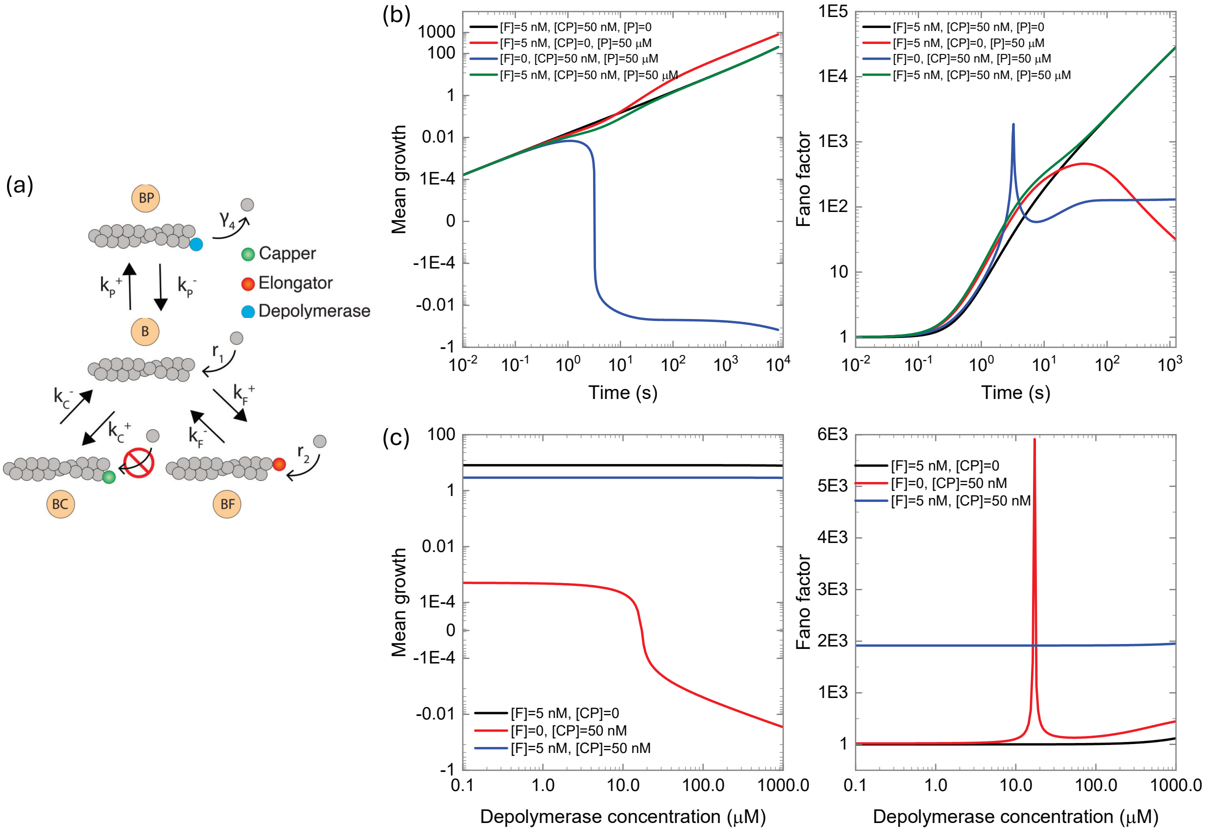

Figure B: **Combined effect of an elongator, a capper and a depolymerase on actin filament length.** (a) Competitive model is illustrated. (b) Mean filament growth (μm) and Fano factor are plotted as a function of time for varying elongator, capper and depolymerase concentrations. (c) long-time mean growth (μm) and Fano factor are shown as functions of depolymerase concentration for different concentrations of elongator and capper. To convert the mean growths from subunits to μm, we use 1 subunit $\sim 0.0027 \mu m$ [4]. However, we keep Fano factor in the unit of subunits of actin monomers. Following parameters are used for elongator (formin) and capper: $r_1 = 6$ subunits/s, $r_2 = 30$ subunits/s, $\widetilde{k}_F^+ = 29.1\, \mu M^{-1} s^{-1}$, and $k_F^- = 8.1 \times 10^{-5}$ s^{-1} [4]. The parameters for depolymerase are: $\gamma_4 = 6.6$ subunits/s, $\widetilde{k}_P^+ = 10^{-2}\, \mu M^{-1} s^{-1}$, and $k_P^- = 0.187$ s^{-1}. The binding and unbinding rates are evaluated based on $K_D = 18.7 \mu M$, reported in [5].

rate ω (equal forward and backward rates).

On the other hand, we derive the moments in long-time limit within the time window τ using Eq. (18). The mean and variance are given by,

$$\langle \Delta L_\tau \rangle = 0, \quad (53)$$

$$\sigma^2_{\Delta L_\tau} = \omega\tau - \frac{\omega^2}{2k^2}\left(1 - 2kt - e^{-2kt}\right). \quad (54)$$

The vanishing mean reflects that state equilibrium leads to equal probabilities for both polymerization and depolymerization, resulting in no net growth. The variance expression contains two contributions: a linear term in τ representing diffusive spreading, and a correction term arising from state switching. In the fast-switching limit ($k \to \infty$), the variance simplifies to $\sigma^2_{\Delta L_\tau} = \omega\tau$, which corresponds to effective diffusive dynamics of a symmetric random walk with net jump rate ω.

6 A three-regulator system of actin dynamics

Here we show an example for three-regulator system– elongator, capper, and depolymerase for actin length regulation. The depolymerase, such as profilin, binds to the barbed end to increase the rate of depolymerization. However, profilin has a significantly higher affinity ($\sim$ 100 fold) for actin monomers compared to the free barbed end [6, 7]. As a result, most of the free monomers within cells are profilin-bound. A high concentrations of profilin were found to result in shortening of actin filaments in lamellipodia [8, 9]. Keeping this in mind, we model the binding-unbinding

kinetics of profilin to the barbed end by considering the dissociation constant of $K_D = 18.7\mu M$, reported in [5].

In presence of the three regulators, namely– formin (elongator), capping protein (capper), and profilin, the filament can exist in four states, representing a competitive model. These are B, BF, BC, and BP, which corresponds to free, elongator-bound, capper-bound, and depolymerase-bound barbed end, respectively (Fig Ba). The respective rate of polymerization from B, BF are r_1 and r_2, while at BC $r_3 = 0$. The rate of depolymerization at BP is γ_4. The binding rates of elongator, capper and depolymerase are denoted by $k_F^+ = \widetilde{k}_F^+[F]$, $k_C^+ = \widetilde{k}_C^+[C]$, and $k_P^+ = \widetilde{k}_P^+[P]$, respectively. The corresponding unbinding rates are k_F^-, k_C^-, and k_P^-. The master equation for this model is given by Eq. (1) given in the main text, where,

$$\mathbf{P}(\Delta L_t) = \begin{pmatrix} P_B(\Delta L_t) \\ P_{BF}(\Delta L_t) \\ P_{BC}(\Delta L_t) \\ P_{BP}(\Delta L_t) \end{pmatrix}, \hat{\mathbf{K}} = \begin{pmatrix} -(k_F^+ + k_C^+ + k_P^+) & k_F^- & k_C^- & k_P^- \\ k_F^+ & -k_F^- & 0 & 0 \\ k_C^+ & 0 & -k_C^- & 0 \\ k_P^+ & 0 & 0 & -k_P^- \end{pmatrix}, \text{and } \hat{\mathbf{R}} = \begin{pmatrix} r_1 & 0 & 0 & 0 \\ 0 & r_2 & 0 & 0 \\ 0 & 0 & 0 & 0 \\ 0 & 0 & 0 & \gamma_4 \end{pmatrix}.$$

Using Eq. (13), we derive the corresponding moments $\langle \Delta L_t \rangle$, and $\langle \Delta L_t^2 \rangle$. The variance, then, can be written as $\sigma^2_{\Delta L_t} = \langle \Delta L_t^2 \rangle - \langle \Delta L_t \rangle^2$. Due to the complex analytical expressions, we don't report them here. The temporal variation of mean growth and Fano factor are shown in Fig Bb. These profiles show the interplay between the three regulators. Perhaps, when elongator is absent, the depolymerization from BP dominates over polymerization from B. In this scenario, the nature of mean growth and Fano factor show notable characteristics. Specifically, the Fano factor shows a sharp peak in this case, caused by a sudden shift in the mean growth behavior.

Next, we calculate the mean and variance in long-time limit from the moments $\langle \Delta L_\tau \rangle$ and $\langle \Delta L_\tau^2 \rangle$ calculated using Eq. (18). The closed form expressions of mean and variance are not reported here because of their complex expressions. The variation in mean growth and Fano factor are plotted as a function of depolymerase concentration (Fig Bc). Again the mean growth and Fano factor have characteristic variation when elongator is absent. Specifically, the Fano factor exhibits a sharp peak due to an abrupt transition in the mean growth trend.

7 Error analysis for sample mean and variance

To quantify the uncertainty in sample-based estimates of the mean, variance, and Fano factor of filament length changes, we derive expressions for the relative errors associated with these quantities. The derivation assumes that the samples are statistically independent, with finite mean $\mu \equiv \langle \Delta L \rangle$ and variance $\sigma^2 \equiv \sigma^2_{\Delta L}$.

Let us consider a sample size of N with sample mean $\hat{\mu}$. Assuming that the individual measurements do not influence each other and share the same mean and variance, the expected value and variance of the sample mean are given by,

$$\mathbb{E}[\hat{\mu}] = \mu, \tag{55}$$

$$\mathrm{Var}[\hat{\mu}] \sim \frac{\sigma^2}{N}. \tag{56}$$

Thus, the relative standard error in the sample mean is,

$$\frac{\Delta\hat{\mu}}{\mu} = \frac{\sqrt{\mathrm{Var}[\hat{\mu}]}}{\mathbb{E}[\hat{\mu}]} = \sqrt{\frac{F}{N\mu}}, \tag{57}$$

where the finite Fano factor is defined as $F = \sigma^2/\mu$.

For the given sample size, let the sample variance be $\hat{\sigma}^2$. Assuming that the scaled sample variance $((N-1)\hat{\sigma}^2/\sigma^2)$ follows a chi-squared distribution with $N-1$ degrees of freedom, the expected value and variance of $\hat{\sigma}^2$ become,

$$\mathbb{E}[\hat{\sigma}^2] = \sigma^2, \tag{58}$$

$$\mathrm{Var}[\hat{\sigma}^2] = \frac{2\sigma^4}{N-1} \sim \frac{2\sigma^4}{N}. \tag{59}$$

The relative error in sample variance is,

$$\frac{\Delta\hat{\sigma}^2}{\sigma^2} = \frac{\sqrt{\mathrm{Var}[\hat{\sigma}^2]}}{\mathbb{E}[\hat{\sigma}^2]} = \sqrt{\frac{2}{N}}. \tag{60}$$

The sample Fano factor is defined as $\hat{F} = \hat{\sigma}^2/\hat{\mu}$. Assuming that the samples are independent, the variance of the sample Fano can be calculated using standard error propagation as,

$$\begin{aligned}\text{Var}[\hat{F}] &= \left(\frac{\partial \hat{F}}{\partial \hat{\sigma}^2}\right)^2 \text{Var}[\hat{\sigma}^2] + \left(\frac{\partial \hat{F}}{\partial \hat{\mu}}\right)^2 \text{Var}[\hat{\mu}], \\ &= \frac{1}{\hat{\mu}^2}\text{Var}[\hat{\sigma}^2] + \frac{\hat{\sigma}^4}{\hat{\mu}^4}\text{Var}[\hat{\mu}]. \end{aligned} \tag{61}$$

The relative error in $\hat{F}$ becomes,

$$\begin{aligned}\epsilon^2 = \left(\frac{\Delta \hat{F}}{F}\right)^2 &= \frac{\text{Var}[\hat{F}]}{\mathbb{E}[\hat{F}]}, \\ &\sim \frac{\text{Var}[\hat{\sigma}^2]}{\hat{\sigma}^4} + \frac{\text{Var}[\hat{\mu}]}{\hat{\mu}^2}, \\ &\sim \left(\frac{\Delta\hat{\sigma}^2}{\sigma^2}\right)^2 + \left(\frac{\Delta\hat{\mu}}{\mu}\right)^2, \\ &\sim \frac{2}{N} + \frac{F}{N\mu} = \frac{1}{N}\left(2 + \frac{F}{\mu}\right). \end{aligned} \tag{62}$$

For the given relative error ϵ, the required sample size is,

$$N \sim \frac{1}{\epsilon^2}\left(2 + \frac{F}{\mu}\right). \tag{63}$$

Note that the required sample size is influenced not only by the absolute value of the Fano factor but also by the mean, as both contribute to the overall estimation error. Here, both F and μ are expressed in the unit of actin monomer subunits. However, to use the mean value in μm unit, we use the relation 1 subunit $\approx 0.0027\mu m$ [4], which gives,

$$N \sim \frac{1}{\epsilon^2}\left(2 + 0.0027 \times \frac{F}{\mu}\right), \tag{64}$$

where F is in subunits and μ is in μm.

8 Experimental data analysis

The experimental dataset analyzed in this work (Fig. 6) has previously been published in Shekhar et al. [4]. In this experiment, actin filaments with free barbed ends were elongated from coverslip-anchored spectrin-actin seeds in the presence of profilin and G-actin. These filaments were then exposed to a solution containing formin mDia1, profilin, and G-actin. This led to a gradual reduction in the fraction of filaments elongating with free barbed ends and an increase in the fraction of filaments elongating with formin at their barbed ends over time. Changes in filament length over time were recorded using microfluidics-assisted TIRF (mF-TIRF) imaging [10]. From these images, we extracted length-versus-time trajectories for 37 individual filaments using kymograph analysis in ImageJ. For each filament trajectory, the time axis was shifted such that the initial time point corresponds to $t = 0$, i.e., all time values were computed relative to the starting time of that filament. Similarly, filament length at each time point was measured relative to the initial length, yielding the net change in length over time. Based on the extracted length data, we computed the sample Fano factor at each time point, defined as the ratio of the sample variance to the sample mean of filament lengths. To estimate the uncertainty in the Fano factor, we employed a bootstrap resampling method over the filament population at each time point.

For theoretical comparison, we computed the Fano factor predicted by the two-state model of actin length regulation by an elongator using the following parameter set: $r_1 = 6$ subunits/s, $r_2 = 16$ subunits/s, $\widetilde{k}_F^+ = 8.8$ $\mu\text{M}^{-1}\text{s}^{-1}$, $k_F^- = 14.7 \times 10^{-5}$ s^{-1}, and $[F] = 5$ nM. The parameter set was chosen to qualitatively reproduce the experimental data and their values are comparable to those reported in [4].

The resulting comparison between the experimental Fano factor and the theoretical prediction is shown in Fig 6. The respective Python script is available in the GitHub repository linked under *Data availability statement.*

References

[1] N G van Kampen. *Stochastic Processes in Physics and Chemistry, 3rd ed.* North-Holland, Amsterdam, 2007.

[2] A. Sánchez and J. Kondev. Transcriptional control of noise in gene expression. *Proc. Natl. Acad. Sci., USA*, 105:5081–5086, 2008.

[3] S. Choubey. Nascent rna kinetics: Transient and steady state behavior of models of transcription. *Phys. Rev. E*, 97:022402, 2018.

[4] S. Shekhar, M. Kerleau, S. Kühn, J. Pernier, G. Romet-Lemonne, A. Jégou, and M. F. Carlier. Formin and capping protein together embrace the actin filament in a ménage à trois. *Nat. Commun.*, 6:8730, 2015.

[5] Ankita Arya, Sandeep Choubey, and Shashank Shekhar. Actin filament barbed-end depolymerization by combined action of profilin, cofilin, and twinfilin. *PRX Life*, 2:033002, Jul 2024.

[6] H. J. Kinosian, L. A. Selden, L. C. Gershman, and J. E. Estes. Interdependence of profilin, cation, and nucleotide binding to vertebrate non-muscle actin. *Biochemistry*, 39:13176–13188, 2000.

[7] A. Jégou, T. Niedermayer, J. Orbán, D. Didry, R. Lipowsky, M. F. Carlier, and G. Romet-Lemonne. Individual actin filaments in a microfluidic flow reveal the mechanism of ATP hydrolysis and give insight into the properties of profilin. *PLoS Biol.*, 9:e1001161, 2011.

[8] M. E. Joy, L. L. Vollmer, K. Hulkower, A. M. Stern, C. K. Peterson, R. C. Boltz, P. Roy, and A. Vogt. A high-content, multiplexed screen in human breast cancer cells identifies profilin-1 inducers with anti-migratory activities. *PLoS One*, 9:e88350, 2014.

[9] J. D. Rotty, C. Wu, E. M. Haynes, C. Suarez, J. D. Winkelman, H. E. Johnson, J. M. Haugh, D. R. Kovar, and J. E. Bear. Profilin-1 serves as a gatekeeper for actin assembly by Arp2/3-dependent and -independent pathways. *Dev. Cell*, 32:54–67, 2015.

[10] S. Shekhar. Microfluidics-assisted tirf imaging to study single actin filament dynamics. *Curr. Protoc. Cell Biol.*, 77(1):12.13.1–12.13.24, 2017.
