# Supplementary material for: A generalized theoretical framework to investigate multicomponent actin dynamics
Source: PLoS Comput Biol. 2025 Sep 8;21(9):e1013434. doi: 10.1371/journal.pcbi.1013434 (PMC12431664; doi:10.1371/journal.pcbi.1013434)
Supplement: Attachment — Submitted filename: nandi_etal_plos.pdf [file pcbi.1013434.s002.pdf]

March 19, 2025

The manuscript studies a general model of actin filament kinetics, including growth and regulatory protein binding within a unified framework. This work addresses an important topic, as there has been significant interest in theories of biofilament length regulation recently, and the examples provided help to demonstrate a promising general approach. I feel this work needs to be developed further before it is suitable for publication. In particular, the connection to biology should be strengthened; the model suggests clear qualitative trends in the filament length distributions vs. regulatory protein concentrations, but there is no attempt to compare the model results to data. The calculations themselves are interesting, though I found the terminology confusing at times and have some further questions, below.

I have the following questions and comments:

(i) Is there a better terminology than "the steady-state distribution of changes in length" to something clearer? It's confusing to call this a steady-state when the filament length distributions themselves are time-dependent. I understand that the authors are referring to the steady-state distribution of filament states, but this should be more clearly distinguished from the distribution of length changes

(ii) Some details of the calculations are left out, e.g. it is stated that "the computation of these two vectors has been performed using suitable software" but the details are not included. Providing code (e.g. Mathematica) with the full representative calculations would help make the manuscript more self-contained and reproducible

(iii) In (S1) to (S3), I did not see where the passage from infinite sum to a finite sum is justified. How do the authors deal with this issue in practice, since the filaments are growing without bound?

(iv) Are the calculations on filament state dynamics equivalent to computing the steady-state from a standard Markov matrix? If so, the results should be connected to the standard formulas for identifying the steady-state vector of a Markov chain

(v) The present model is quite abstract and does not provide any comparison to biological data. Attempting to compare to some experimental data on filament length distributions and resulting fano factors, at least in some special case, would provide important motivation and help make the work more concrete

(vi) Are there scaling relationships that yield a collapse of some of the data (e.g. fano factor vs concentration in Figs 2, 3, 4)? Given the analytical formulas for fano factor this seems like it should be possible but it does not appear to have been explored
