# Supplementary material for: A generalized theoretical framework to investigate multicomponent actin dynamics
Source: PLoS Comput Biol. 2025 Sep 8;21(9):e1013434. doi: 10.1371/journal.pcbi.1013434 (PMC12431664; doi:10.1371/journal.pcbi.1013434)
Supplement: Attachment — Submitted filename: NandiChoubeyShekhar_PLoSCompBio_Review.pdf [file pcbi.1013434.s003.pdf]

In this manuscript, Nandi et al present a theoretical framework for modeling actin filament dynamics under the regulation of multiple actin-binding proteins (ABPs). By deriving closed-form expressions for filament growth distributions, the study aims to provide a generalizable approach to understanding actin regulation. In particular, the authors analytically calculate an experimentally accessible quantity, the Fano factor for growth increments, as a function of time. Fano factor dynamics and dependencies on ABP concentrations can then be presumably compared to experimental data to perform model selection and thus identify the mechanism underlying the regulation of actin length. Specifically, the authors apply their model to distinguish between alternative models of capper and elongator binding to actin. In their analysis, the authors observe distinct Fano factor responses to competitive versus simultaneous mechanisms of capper and elongator binding.

The mathematical model itself is simple and the proposed use of it elegant. The analysis to achieve analytical results looks fearsome at times, but we saw no obvious errors in the analysis except for a point of confusion I highlight below.

Major Points

Strengths:

The two major strengths of this study are:

1. Ease of comparison to experiment: The most important application of the model is to distinguish mechanisms of ABP binding to actin, especially with regard to the nature of the binding events when multiple ABP's are present simultaneously. These differing mechanisms are difficult to directly assay in experiment and so fluctuation-based inference is a valuable tool to use more readily available data (i.e., actin polymer length *change* distributions even given an arbitrary starting timepoint from the point of view of a given observed filament, we appreciate that it was very clever to think of a way to use this variable instead of having to track a given filament length over time continuously) to decipher mechanism. The clearly distinct patterns in the Fano factor traces make it potentially possible to achieve identifiability of a given ABP binding mechanism: differing mechanisms yield distinct Fano factor dynamics and the framework could be useful for model identifiability.
2. Analytical control over the mathematical framework, again easing comparison to experiment: Rather than settle for simply simulating the model, we believe that the analytical expressions derived here are especially useful as they allow rapid comparison of experiment and theory without the need to search a large, possibly ill-constrained parameter space and carry out cumbersome simulations for each set of parameters chosen.

Weaknesses:

1. Tau window analysis: We don't think we understand the meaning of the steady state distribution within a fixed tau window, at least as described in the main text. What time is being taken to infinity so that the distribution settles down to steady state and how is that time related to the time window tau? This seems to be a central point in the analysis so

any reader who shares our confusion may not be able to successfully build upon this work.

2. Potential for implausibly high experimental data requirements to perform comparison between model and experiment: One question for the authors is whether the actual numerical values for the Fano factors presented in the manuscript are to be taken literally. In short, these Fano factors are gigantic (order 10^3). One concern with the magnitude of the Fano factors, should their numerical values as presented actually be taken seriously, is that it calls into question whether comparison to experimental data will be feasible. Specifically, how much data would one need to take to capture a distribution whose variance is several thousand fold bigger than the mean? Consider the following back-of-the-envelope estimate of how the data requirements would scale with the magnitude of the Fano factor:

   To estimate the error of the Fano factor:

$$F = \frac{\sigma^2}{\mu}$$

   For a Gaussian or Poisson-like variable, the **variance of the sample variance** scales as:

$$\mathrm{Var}[\hat{\sigma}^2] \sim \frac{2\sigma^4}{N}$$

   The relative error

$$\frac{\Delta\hat{\sigma}^2}{\sigma^2} \sim \sqrt{\frac{2}{N}}$$

   The **variance of the sample mean** is:

$$\mathrm{Var}[\hat{\mu}] \sim \frac{\sigma^2}{N}$$

   The relative error

$$\frac{\Delta\hat{\mu}}{\mu} \sim \frac{\sigma}{\mu}\frac{1}{\sqrt{N}} = \sqrt{\frac{F}{N}}$$

   The propagation from both errors is

$$\left(\frac{\Delta F}{F}\right)^2 \sim \left(2 \cdot \frac{\Delta\hat{\sigma}^2}{\hat{\sigma}^2}\right)^2 + \left(\frac{\Delta\hat{\mu}}{\hat{\mu}}\right)^2 \sim 4 \cdot \frac{2}{N} + \frac{F}{N}$$

   When F ~ 1000 >> 1, the error will be dominated by the 2nd term.

$$\frac{\Delta F}{F} \sim \sqrt{\frac{F}{N}}$$

   For a given relative error ε, the required sample size N:

$$N \sim F \cdot \frac{1}{\epsilon^2}$$

   Thus for even relatively poor relative precision, according to this simple analysis the number of samples required to achieve such a precision could run well into the range of $>10^3$ to 10^5 if the Fano factors are of order 10^3. If we have made an error in this

estimate of required data we certainly apologize in advance. But even if we made a mistake, a correct version of this type of analysis from the authors would go a long way toward demonstrating the authors' key claim that this analysis tool can be used in conjunction with experiment to perform model selection. On the other hand if we did not make a mistake, then perhaps this tool is not as easy to compare to experiment as it seems upon first reading or the authors need to make a case that collecting 10's of thousands of actin growth tracks is plausible.

3. Lack of comparison to experiment (related to point above): In the case that our feedback about the data requirement estimate mentioned above is incorrect, another way to demonstrate this is by comparison to already collected data. Even if data that dissects out capper versus elongator effects on actin growth are not available, even showing that the kinds of Fano factors expected theoretically are observed in the same ballpark experimentally (including the requisite error analysis to make sure that the large Fano factor is not a result due to domination by measurement noise) would be an important way to bolster these mathematical results.

Minor Points

1. The introduction to ΔL_t uses confusing wording: The authors write that "ΔL_t can be positive (polymerization) or negative (depolymerization)" but if we understand this correctly, ΔL_t can be positive for a given filament even if it is in the process of depolymerizing. Apologies if we are in error, but our interpretation of the definition is that positive ΔL_t just means that the length of the filament at time t is greater than it was at t = 0 but doesn't say whether it is polymerizing or depolymerizing.
2. Intuitive explanation of trends of Fano factor versus ABP concentration: The presentation would be improved if the authors would give a more intuitive explanation of the Figs 2 and 3 results where the Fano factor goes to 1 at both extremes. For example, the authors write: "The increase at intermediate concentrations is due to filaments switching between the B and BF states." One more sentence about why it is 1 at the extremes (for example, for the elongator case in Fig. 2 presumably this is because
3. Very minor (certainly not determinative of the publication-worthiness of the manuscript): the lines of the Fano factor plots are too thin, the colors are hard to see, and the schematic in Fig. 1 could do a clearer job of illustrating the various variables and quantities in the model (especially t versus tau, but also the various polymerizing versus depolymerizing states, maybe even a schematic of the master equation with an illustration of the K and R matrices).

We thank the authors for sharing this interesting approach to achieving mechanistic insight into actin dynamics and hope they find our feedback useful.
