# Supplementary material for: A generalized theoretical framework to investigate multicomponent actin dynamics
Source: PLoS Comput Biol. 2025 Sep 8;21(9):e1013434. doi: 10.1371/journal.pcbi.1013434 (PMC12431664; doi:10.1371/journal.pcbi.1013434)
Supplement: Attachment — Submitted filename: Response to Reviewers.pdf [file pcbi.1013434.s004.pdf]

We would first like to thank the reviewers for appreciating our study and their valuable comments, which we believe have significantly improved the quality of our manuscript. In particular, we have extensively modified our model section to clarify the points raised by the reviewers. We have now additionally included a comparison of our theory with experimental data. We hope this revised manuscript will be suitable for publication. Below, we reproduce the reviewer comments (in red) and give our responses (in black), including the relevant changes to the manuscript, which were made in response to the specific referee comments. The changes made in the main text of the manuscript are highlighted in blue.

Reviewer 1:

How the multitude of actin binding proteins (ABPs) found in cells contribute to controlling the sizes of actin filaments is an interesting and important question. To address this, the authors present kinetic model of actin filament assembly that considers how these regulatory factors influence both the evolution of filament length over time, as well as the steady-state distribution of filament lengths. They use this model to explore how these aspects of filament length change in the presence of an elongator, a capper, and finally a mixed population of these two regulators. They find that in addition to their influence over the length of filaments, these regulators have different effects on the filament-to-filament variability in length. While this model may present a useful way to distinguish between different regulatory mechanisms of multiple ABPs, there are a number of issues that should be addressed prior to publication.

We would like to thank the reviewer for appreciating our theoretical framework. Below, we provide detailed responses to the reviewer's comments.

Major issues:

1. The description of the model is tough to parse and could use a plain-language description of how this model differs from prior models (especially: https://doi.org/10.1016/j.bpj.2022.05.014 and 10.1146/annurev-biophys-070915-094206). As written, it is hard to assess the novelty of this model.

Response: We apologize for the ambiguity in our earlier phrasing.

Unlike the referenced studies, which focused primarily on steady-state distributions, our kinetic model captures both transient and long-term behavior of actin filament length. Moreover, previous studies dissected the effect of one or two acting binding proteins (ABPs) on filament length. In contrast, we propose a generalized theoretical framework to capture the effect of an arbitrary number of ABPs on filament length. Moreover, we provide exact analytical results of the moments of the filament length distribution over time.

We have now revised both the Introduction and the Model sections for clarity (see lines 10-89).

2. While the authors claim that this model can capture the simultaneous effects of an arbitrary number of ABPs they only explore scenarios that include either single regulators or a pair of regulators. There are many examples of models that consider the effect of 1-2

regulators, so it would be nice to have a demonstration of how this model can be applied to 2+ regulators.

Response: Thank you for the suggestion. Our primary goal in this work is to present a generalized analytical framework capable of capturing the regulatory effects of an arbitrary number of ABPs. While the main text focuses on illustrative examples involving up to two regulators, the model is fully extendable to systems with more than two ABPs. Following the reviewer's suggestion, **we now include an example with 3 regulators in the Supplementary Text to demonstrate the model's broader applicability (see Sec. S6).**

3. The examples used do not appear to include filament disassembly and only consider filaments that have the capacity to grow. Can the authors comment why disassembly is not included in these examples?

Response: Thank you for bringing this to our attention. Our general framework accounts for both polymerization and depolymerization processes. Depolymerization is incorporated through specific filament states that represent disassembly. However, the specific systems we initially analyzed did not explicitly include depolymerization. It's important to note that our model does not explicitly account for polymerization or depolymerization from the barbed or pointed ends, and is agnostic to the precise binding locations of actin-binding proteins (ABPs). Instead, the model assumes that filament growth or shrinkage occurs when the filament is bound to a regulatory protein. To explicitly demonstrate how our model accommodates disassembly, we have now considered two additional models: (1) a one-state depolymerization model, which explores the effect of a single depolymerase on filament length, and (2) a three-regulator model, which investigates the combined effects of three ABPs—one of which functions as a depolymerase—on filament length.

We have added the results of these models in the Supplementary Text (see Sec. S5.1 and S6).

4. In the description of the model the authors mention that capped filament states can be considered as part of the state leading to polymerization. These seem counterintuitive and this assumption could use further discussion.

Response: Thank you for raising this point. In our framework, actin filaments are assumed to exhibit two different classes of filament states, namely polymerizing and depolymerizing. The capped states are included within the polymerization group with zero rate of polymerization. This approach avoids the need to define a separate category for capped states and simplifies the analysis. However, the capped states can also be included within the depolymerizing group without the loss of generality. It provides a consistent and tractable way to model both growing and non-growing filament states within a unified framework. **We have clarified this point in the revised manuscript (see lines 69-72).**

5. In figure 3B, the x-axis in the right panel is truncated compared to the panel on the left. Extending this axis would support the author's claim that the fano factor reaches a plateau in the long-time limit. Currently, the lower concentration of CP would appear to increase.

Response: Thank you for pointing this out. We have extended the X-axis to visually clarify that the Fano factor plateaus in the long-time limit. **We have now modified this figure accordingly in the revised manuscript (see Figure 4B in the revised manuscript).**

6. The results from Figure 4 appear to be the strongest support for their claims. Do the results in 4C support prior models of formin/capping protein behavior where the distribution depends on the concentration of these regulators?

Response: Thank you for the comment. Yes, the results in Fig. 4C are consistent with previous studies demonstrating that filament length distributions depend on the concentrations of formin and capping proteins. Notably, earlier models explained this concentration dependence through a limiting pool mechanism for actin monomers [Mohapatra et al., 2016; Banerjee et al., 2022). Note that our model does not explicitly consider the effect of a finite monomer pool of actin. Despite this difference in approach, our results align with the concentration-dependent regulation reported in prior studies. **We have now clarified this connection in the revised manuscript (see lines 243-246).**

Minor issues:

1. The manuscript should be carefully reviewed as there are a number of typos. Without line numbers it is difficult to point out specific examples.

Response: We apologize for the typos. We have now carefully proofread the manuscript and corrected the typographical errors. **We have added line numbers for convenience in the revised manuscript.**

2. The arrows in the cartoon of Fig 2 appear to be mislabeled.

Response: Thank you for pointing this out. We have reviewed Figure 2 and corrected the mislabeled arrow in the revised manuscript (**see Figure 3 in the revised version**).

3. A clear definition of what 'mean filament growth' means and whether this different than 'length' would be helpful.

Response: Thank you for the comment. We use the term "mean filament growth" to refer to the average change in filament length over time, starting from an initial reference mean

length $< L_0 >$, i.e., $< \Delta L_t >= < L_t > - < L_0 >$, where $< L_t >$ refers to the mean filament length at time t. ΔL_t quantifies the net elongation or shortening due to polymerization and depolymerization processes. **We have clarified this definition in the revised manuscript to avoid confusion with the absolute filament length (see lines 128-133).**

4. Figure 4A – BF and BC are mislabeled, as are the arrows.

Response: Thank you for bringing this to our attention. **We have corrected the labels and arrows in the revised manuscript (see Figure 5A in the revised manuscript).**

5. Figure 4C is not referenced in the manuscript and the figure legend should mention which plot corresponds to which model.

Response: Thank you for pointing this out. **We have now referenced Fig. 4C appropriately in the main text and updated the figure legend to indicate which plot corresponds to which model in the revised manuscript (see legend of Figure 5).**

Reviewer 2:

The manuscript studies a general model of actin filament kinetics, including growth and regulatory protein binding within a unified framework. This work addresses an important topic, as there has been significant interest in theories of biofilament length regulation recently, and the examples provided help to demonstrate a promising general approach. I feel this work needs to be developed further before it is suitable for publication. In particular, the connection to biology should be strengthened; the model suggests clear qualitative trends in the filament length distributions vs. regulatory protein concentrations, but there is no attempt to compare the model results to data. The calculations themselves are interesting, though I found the terminology confusing at times and have some further questions, below. I have the following questions and comments:

We thank the reviewer for their constructive and thoughtful comments. We appreciate the suggestions regarding the biological relevance and clarity of the manuscript. In the revised version, we have addressed all concerns raised and responded in detail to the specific questions and comments provided below.

(i) Is there a better terminology than "the steady-state distribution of changes in length" to something clearer? It's confusing to call this a steady-state when the filament length distributions themselves are time-dependent. I understand that the authors are referring to the steady-state distribution of filament states, but this should be more clearly distinguished from the distribution of length changes

Response: Thank you for the suggestion. We agree that the term "steady-state distribution of changes in length" may be misleading. As the reviewer correctly pointed out, we compute the moments of filament length over a time window τ, assuming the filament states have reached a steady state. **We now refer to this in the revised manuscript as "long-time distribution" for clarity. Moreover, we have clarified this point in the manuscript (lines 39-42 and 116-122).**

(ii) Some details of the calculations are left out, e.g. it is stated that "the computation of these two vectors has been performed using suitable software" but the details are not included. Providing code (e.g. Mathematica) with the full representative calculations would help make the manuscript more self-contained and reproducible

Response: Thank you for the suggestion. We have now included a link to a GitHub repository containing the scripts and representative calculations to ensure the manuscript is more self-contained and reproducible. **The link is provided in the revised manuscript (see Data Availability).**

(iii) In (S1) to (S3), I did not see where the passage from infinite sum to a finite sum is justified. How do the authors deal with this issue in practice, since the filaments are growing without bound?

Response: In Eqs. (S1) and (S2), we define vectors of partial moments where each element involves an infinite sum over all possible values of ΔL_t, reflecting the unbounded nature of filament growth. Equation (S3) is not an infinite sum in itself- it simply sums all the components of the vector defined in Eq. (S2) to yield the n-th central moment. Thus, the infinite summations are already accounted for in the construction of each element of the vectors in Eqs. (S1) and (S2), and Eq. (S3) is a finite sum over those N elements. No truncation is applied in this analytical treatment, the infinite summations are retained as part of the formal derivation. **We now clarify this point in the revised Supplementary text (see the paragraph immediately after Eq. S3).**

(iv) Are the calculations on filament state dynamics equivalent to computing the steady-state from a standard Markov matrix? If so, the results should be connected to the standard formulas for identifying the steady-state vector of a Markov chain

Response: The calculations on filament state dynamics in our model are not fully equivalent to computing the steady state from a standard (discrete-time) Markov matrix, but they are closely analogous. While standard Markov matrices are stochastic matrices used in discrete-time models (where columns sum to one), our framework uses a continuous-time master equation with a rate matrix $\widehat{K}$, where columns sum to zero. At steady state, we solve $\widehat{K}.\overline{\Delta L}^{(0)} = 0$ (see Eq. (S14)), which is analogous to identifying the stationary distribution in Markov chains.

Importantly, our work extends beyond steady-state analysis by computing the full-time evolution of filament length distributions and their statistical moments (see Eqs. S1–S18). This allows us to capture both transient and long-time dynamics under multicomponent regulation, a key distinction from standard Markov chain approaches. **We have added a brief discussion on this in the revised Supplementary text (see the paragraph immediately after Eq. S15).**

(v) The present model is quite abstract and does not provide any comparison to biological data. Attempting to compare to some experimental data on filament length distributions and resulting fano factors, at least in some special case, would provide important motivation and help make the work more concrete

Response: Thank you for pointing out the importance of anchoring our theoretical framework in experimental observations. In response, we have now included a direct comparison between our model predictions and experimental data of actin filament elongation. Specifically, we analyzed a dataset from one of our previous manuscripts (Shekhar et al, 2015 Nat. Commn.), which employed mF-TIRF microscopy-based measurements of filament growth in the presence of formin mDia1. We extracted 37 single-filament growth trajectories from time-dependent image sequences and computed the time-dependent Fano factor based on these 37 filaments.

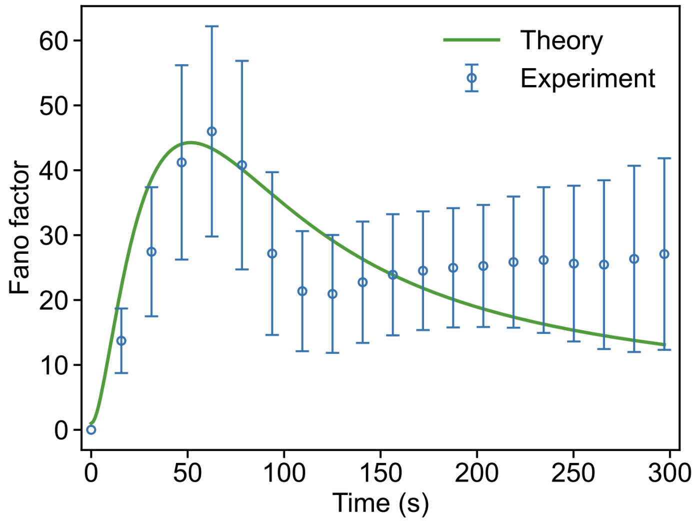

These experimental results are compared with predictions from our two-state elongator model (see Figure 3A) in the revised manuscript. We find that the model captures the qualitative features of the experimental data, including the non-monotonic behavior of the Fano factor at early times and the overall magnitude of variability. However, for a proper comparison between theory and experiments, we will in future design new experiments where the concentration of various regulatory proteins is tuned systematically over a larger number of filaments.

We believe that this comparison between theory and experiments demonstrates the utility of our theoretical framework in gaining mechanistic insights into the regulation of filament length. **This comparison is now presented in Figure 6. We also now discuss this in the revised manuscript (lines 293–311). We have additionally added details on the data extraction and analysis procedure in the revised Supplementary Text (see Section S8).**

(vi) Are there scaling relationships that yield a collapse of some of the data (e.g. fano factor vs concentration in Figs 2, 3, 4)? Given the analytical formulas for fano factor this seems like it should be possible but it does not appear to have been explored

Response: Thank you for your query. The apparent collapse of the Fano factor curves across different concentrations can be explained by the limiting behaviour derived from the analytical expressions. Specifically, the Fano factor approaches 1 at both very low and very high regulator concentrations. These convergences arise from the system's effective reduction to a single dominant state under these conditions, thereby minimizing variability. **We now clarify this in the revised manuscript (see lines 173-183 and 206-216).**

Reviewer 3:

In this manuscript, Nandi et al present a theoretical framework for modeling actin filament dynamics under the regulation of multiple actin-binding proteins (ABPs). By deriving closed-form expressions for filament growth distributions, the study aims to provide a generalizable approach to understanding actin regulation. In particular, the authors analytically calculate an experimentally accessible quantity, the Fano factor for growth increments, as a function of time. Fano factor dynamics and dependencies on ABP concentrations can then be presumably compared to experimental data to perform model selection and thus identify the mechanism underlying the regulation of actin length. Specifically, the authors apply their model to distinguish between alternative models of capper and elongator binding to actin. In their analysis, the authors observe distinct Fano factor responses to competitive versus simultaneous mechanisms of capper and elongator binding.

The mathematical model itself is simple and the proposed use of it elegant. The analysis to achieve analytical results looks fearsome at times, but we saw no obvious errors in the analysis except for a point of confusion I highlight below.

Major Points

Strengths:
The two major strengths of this study are:

1. Ease of comparison to experiment: The most important application of the model is to distinguish mechanisms of ABP binding to actin, especially with regard to the nature of the binding events when multiple ABP's are present simultaneously. These differing mechanisms are difficult to directly assay in experiment and so fluctuation-based inference is a valuable tool to use more readily available data (i.e., actin polymer length change distributions even given an arbitrary starting timepoint from the point of view of a given observed filament, we appreciate that it was very clever to think of a way to use this variable instead of having to track a given filament length over time continuously) to decipher mechanism. The clearly distinct patterns in the Fano factor traces make it potentially possible to achieve identifiability of a given ABP binding mechanism: differing mechanisms yield distinct Fano factor dynamics and the framework could be useful for model identifiability.

2. Analytical control over the mathematical framework, again easing comparison to experiment: Rather than settle for simply simulating the model, we believe that the analytical expressions derived here are especially useful as they allow rapid comparison of experiment and theory without the need to search a large, possibly ill-constrained parameter space and carry out cumbersome simulations for each set of parameters chosen.

Response: Thank you for your encouraging comments. We appreciate the recognition of our analytical framework and its potential utility in distinguishing ABP regulatory mechanisms. We have revised the manuscript accordingly, addressing all the comments and suggestions, and provided our point-by-point responses below.

Weaknesses:

1. Tau window analysis: We don't think we understand the meaning of the steady state distribution within a fixed tau window, at least as described in the main text. What time is being taken to infinity so that the distribution settles down to steady state and how is that time related to the time window tau? This seems to be a central point in the analysis so any reader who shares our confusion may not be able to successfully build upon this work.

Response: We clearly should have been more careful while defining the steady-state filament length. In fact, another reviewer also pointed this out. Based on the reviewer's comments, we no longer use the term *steady state* and instead refer to the regime as *long-time dynamics* in the revised manuscript, as it better reflects the description of this regime.

It must be noted that the long-time dynamics allude to the scenario whereby the time derivative of the state occupancy probabilities vanishes (the left-hand side of Eq. (S9) is set to zero). This ensures that the underlying state-switching dynamics have attained steady-state. Once this stationarity condition is satisfied, we introduce a fixed time window τ over which we measure the statistical moments (e.g., mean, variance) of changes in filament lengths. The window τ represents an observation period that begins at some arbitrary, but sufficiently large time t, at which the system is assumed to have reached steady-state.

We have clarified this interpretation in the revised manuscript (see lines 39-42 and 116-122).

2. Potential for implausibly high experimental data requirements to perform comparison between model and experiment: One question for the authors is whether the actual numerical values for the Fano factors presented in the manuscript are to be taken literally. In short, these Fano factors are gigantic (order 10^3). One concern with the magnitude of the Fano factors, should their numerical values as presented actually be taken seriously, is that it calls into question whether comparison to experimental data will be feasible. Specifically, how much data would one need to take to capture a distribution whose variance is several thousand fold bigger than the mean? Consider the following back-of-the-envelope estimate of how the data requirements would scale with the magnitude of the Fano factor:

To estimate the error of the Fano factor:

$$F = \frac{\sigma^2}{\mu}$$

For a Gaussian or Poisson-like variable, the variance of the sample variance scales as:

$$Var[\hat{\sigma}^2] \sim \frac{2\sigma^4}{N}$$

The relative error

$$\frac{\Delta\hat{\sigma}^2}{\sigma^2} \sim \sqrt{\frac{2}{N}}$$

The variance of the sample mean is:

$$Var[\hat{\mu}] \sim \frac{\sigma^2}{N}$$

The relative error

$$\frac{\Delta\hat{\mu}}{\mu} \sim \frac{\sigma}{\mu}\frac{1}{\sqrt{\mu}} = \sqrt{\frac{F}{N}}$$

The propagation from both errors is

$$\left(\frac{\Delta F}{F}\right)^2 \sim \left(2 \cdot \frac{\Delta\hat{\sigma}^2}{\hat{\sigma}^2}\right)^2 + \left(\frac{\Delta\hat{\mu}}{\hat{\mu}}\right)^2 \sim 4 \cdot \frac{2}{N} + \frac{F}{N}$$

When F ~ 1000 >> 1, the error will be dominated by the 2nd term.

$$\frac{\Delta F}{F} \sim \sqrt{\frac{F}{N}}$$

For a given relative error ϵ, the required sample size N:

$$N \sim F \cdot \frac{1}{\epsilon^2}$$

Thus for even relatively poor relative precision, according to this simple analysis the number of samples required to achieve such a precision could run well into the range of $> 10^3$ to 10^5 if the Fano factors are of order 10^3. If we have made an error in this estimate of required data we certainly apologize in advance. But even if we made a mistake, a correct version of this type of analysis from the authors would go a long way toward demonstrating the authors' key claim that this analysis tool can be used in conjunction with experiment to perform model selection. On the other hand if we did not make a mistake, then perhaps this tool is not as easy to compare to experiment as it seems upon first reading or the authors need to make a case that collecting 10's of thousands of actin growth tracks is plausible.

Response: Thank you for highlighting this important point. We appreciate the back-of-the-envelope analysis estimating the experimental sample size required for reliable Fano factor estimation. In response, we performed a detailed analytical calculation of the relative errors associated with estimating the sample mean ($\hat{\mu}$) and variance ($\hat{\sigma}^2$), and derived the propagated error in the Fano factor.

The expectation values and variance of the sample mean are given by

$$E[\hat{\mu}] = \mu \quad \text{and} \quad Var[\hat{\mu}] \sim \frac{\sigma^2}{N}$$

Thus, the relative standard error in the sample mean is,

$$\frac{\Delta\hat{\mu}}{\mu} = \frac{\sqrt{Var[\hat{\mu}]}}{E[\hat{\mu}]} = \sqrt{\frac{F}{N\mu}}.$$

Again, assuming that the scaled sample variance $(N-1)\hat{\sigma}^2/\sigma^2$ follows a chi-squared distribution with N-1 degrees of freedom, the expected value and variance of the sample variance can be written as

$$E[\hat{\sigma}^2] = \sigma^2 \quad \text{and} \quad Var[\hat{\sigma}^2] = \frac{2\sigma^4}{N-1} \sim \frac{2\sigma^4}{N}$$

The relative error in sample variance is

$$\frac{\Delta\hat{\sigma}^2}{\sigma^2} = \frac{\sqrt{Var[\hat{\sigma}^2]}}{E[\hat{\sigma}^2]} \sim \sqrt{\frac{2}{N}}$$

Defining the sample Fano factor as $\hat{F} = \hat{\sigma}^2/\hat{\mu}$, we can write the variance using standard error propagation as

$$Var[\hat{F}] = \left(\frac{\partial \hat{F}}{\partial \hat{\sigma}^2}\right)^2 Var[\hat{\sigma}^2] + \left(\frac{\partial \hat{F}}{\partial \hat{\mu}}\right)^2 Var[\hat{\mu}] = \frac{1}{\hat{\mu}^2} Var[\hat{\sigma}^2] + \frac{\hat{\sigma}^4}{\hat{\mu}^4} Var[\hat{\mu}]$$

The relative error is

$$\epsilon^2 = \left(\frac{\Delta\hat{F}}{F}\right)^2 = \frac{Var[\hat{F}]}{E[\hat{F}]} \sim \frac{Var[\hat{F}]}{\hat{F}} \sim \frac{1}{\hat{\sigma}^4} Var[\hat{\sigma}^2] + \frac{1}{\hat{\mu}^2} Var[\hat{\mu}] \sim \frac{1}{N}\left(2 + \frac{F}{\mu}\right)$$

Note that the required sample size is influenced not only by the absolute value of the Fano factor but also by the mean, as both contribute to the overall estimation error. Here, both F and μ are expressed in the unit of actin monomer subunits. However, to use the mean value in μm unit, we use the relation 1 subunit ≈ 0.0027μm,, which gives,

$$N \sim \frac{1}{\epsilon^2}\left(2 + 0.0027 \times \frac{F}{\mu}\right)$$

Where F is in subunits and μ is in μm.

This analysis shows that even for large Fano factor values, the required sample size remains experimentally feasible. For instance, even when F ~ 1000 and μ ~ 10μm, the expression reduces to N ~ $(1/\epsilon^2) \times 2$. Thus, for a 10% relative error ($\epsilon = 0.1$), a sample size of N ~ 200 would be sufficient. This analysis supports the practical utility of our model for experimental comparison and model selection.

These calculations have been incorporated into the revised Supplementary Text (see Sec. S7), which provides the full derivation of the error estimates. A summary of this analysis is also included in the main manuscript (see lines 280–292) to highlight its implications for experimental feasibility and model validation.

3. Lack of comparison to experiment (related to point above): In the case that our feedback about the data requirement estimate mentioned above is incorrect, another way to demonstrate this is by comparison to already collected data. Even if data that dissects out

capper versus elongator effects on actin growth are not available, even showing that the kinds of Fano factors expected theoretically are observed in the same ballpark experimentally (including the requisite error analysis to make sure that the large Fano factor is not a result due to domination by measurement noise) would be an important way to bolster these mathematical results.

Response: Thank you for this important suggestion. To address this point, we analyzed in vitro experimental data from one of our previously published manuscripts (Shekhar et al, 2015 Nat. Commun.). In this specific dataset, actin filaments were grown in the presence of a low concentration of formin mDia1, and filament elongation was recorded over time using mf-TIRF microscopy. From this time-lapse data, we measured filament length trajectories of 37 individual filaments over time. Based on these trajectories, we computed the Fano factor of length change across filaments at each time point.

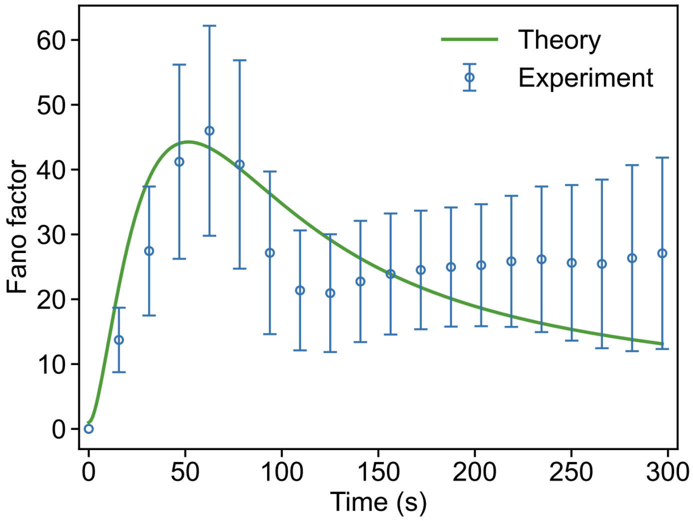

We compared these experimental measurements with theoretical predictions from our two-state elongator model (see Figure 3A). **This comparison is now included in the revised manuscript (see Figure 6).** We find that the model qualitatively captures key features of the experimental Fano factor, including its non-monotonic behavior and the overall magnitude of fluctuations throughout the observed time window. This agreement indicates that the Fano factor predicted by our model is consistent with the experimentally estimated Fano factor obtained from a sample size of ~37 filaments. The bootstrap-estimated error bars reflect statistical uncertainty due to the modest sample size. This analysis demonstrates the utility of our theoretical framework in extracting mechanistic insights from experiments that measure actin filament length as a function of time.

We have explicitly included this comparison in the revised manuscript (see lines 293–311) and provided a detailed description of the experimental data analysis in the revised Supplementary Text (see Section S8).

Minor Points:

1. The introduction to ΔL_t uses confusing wording: The authors write that "ΔL_t can be positive (polymerization) or negative (depolymerization)" but if we understand this correctly, ΔL_t can be positive for a given filament even if it is in the process of depolymerizing. Apologies if we are in error, but our interpretation of the definition is that positive ΔL_t just means that the length of the filament at time t is greater than it was at t =0 but doesn't say whether it is polymerizing or depolymerizing.

Response: We apologize for the confusion caused by our phrasing. A positive value of ΔL_t simply indicates that, over the interval from t=0 to time t, polymerization events have dominated over depolymerization, resulting in net growth. Conversely, a negative ΔL_t indicates that depolymerization has outpaced polymerization in that time window. However, these values do not directly reflect the instantaneous direction of filament dynamics at any given moment. **We have now revised the manuscript to clarify this point (see lines 64-68). To further support this interpretation, we have included a simple one-state depolymerization model in the revised Supplementary Text (see Sec. S5.1).**

2. Intuitive explanation of trends of Fano factor versus ABP concentration: The presentation would be improved if the authors would give a more intuitive explanation of the Figs 2 and 3 results where the Fano factor goes to 1 at both extremes. For example, the authors write: "The increase at intermediate concentrations is due to filaments switching between the B and BF states." One more sentence about why it is 1 at the extremes (for example, for the elongator case in Fig. 2 presumably this is because

Response: Thank you for the suggestion. At both low and high ABP concentrations, the system effectively reduces to a single dominant state, either unbound or fully bound, leading to Poisson-like dynamics with Fano factor approaching 1. This convergence can also be derived directly from the limiting forms of the analytical expressions. **We have incorporated both the intuitive and mathematical explanations in the revised manuscript (see lines 173–183 and 206–216).**

3. Very minor (certainly not determinative of the publication-worthiness of the manuscript): the lines of the Fano factor plots are too thin, the colors are hard to see, and the schematic in Fig. 1 could do a clearer job of illustrating the various variables and quantities in the model (especially t versus tau, but also the various polymerizing versus depolymerizing states, maybe even a schematic of the master equation with an illustration of the K and R matrices).

We thank the authors for sharing this interesting approach to achieving mechanistic insight into actin dynamics and hope they find our feedback useful.

Response: Thank you for the helpful suggestions and encouraging remarks. In response, we have revised Fig. 1 to distinguish between transient and long-time dynamics, and clarify the distinction between t and τ. Additionally, we have introduced a new schematic (now Fig. 2) that explicitly represents the key variables and transitions in a minimal model, including

polymerizing and depolymerizing states, and the structure of the corresponding master equation via the K and R matrices.

We have revised the figure presentation and schematic clarity in the revised manuscript (see Figures 1 and 2 in the revised version).
